# Ncs2* mediates *in vivo* virulence of pathogenic yeast through sulphur modification of cytoplasmic transfer RNA

**DOI:** 10.1093/nar/gkad564

**Published:** 2023-07-18

**Authors:** Fiona Alings, Karin Scharmann, Cristian Eggers, Bettina Böttcher, Mikołaj Sokołowski, Ekaterina Shvetsova, Puneet Sharma, Joël Roth, Leon Rashiti, Sebastian Glatt, Sascha Brunke, Sebastian A Leidel

**Affiliations:** Max Planck Research Group for RNA Biology, Max Planck Institute for Molecular Biomedicine, Muenster, Germany; Max Planck Research Group for RNA Biology, Max Planck Institute for Molecular Biomedicine, Muenster, Germany; Max Planck Research Group for RNA Biology, Max Planck Institute for Molecular Biomedicine, Muenster, Germany; Department of Chemistry, Biochemistry and Pharmaceutical Sciences, University of Bern, Bern, Switzerland; Graduate School for Cellular and Biomedical Sciences, University of Bern, Bern, Switzerland; Department of Microbial Pathogenicity Mechanisms, Leibniz Institute for Natural Product Research and Infection Biology - Hans Knoell Institute, Jena, Germany; Septomics Research Center, Friedrich Schiller University and Leibniz Institute for Natural Product Research and Infection Biology - Hans Knoell Institute, Jena, Germany; Max Planck Research Group at the Malopolska Centre of Biotechnology, Jagiellonian University, Krakow, Poland; Department of Chemistry, Biochemistry and Pharmaceutical Sciences, University of Bern, Bern, Switzerland; Graduate School for Cellular and Biomedical Sciences, University of Bern, Bern, Switzerland; Max Planck Research Group for RNA Biology, Max Planck Institute for Molecular Biomedicine, Muenster, Germany; Department of Chemistry, Biochemistry and Pharmaceutical Sciences, University of Bern, Bern, Switzerland; Department of Chemistry, Biochemistry and Pharmaceutical Sciences, University of Bern, Bern, Switzerland; Department of Chemistry, Biochemistry and Pharmaceutical Sciences, University of Bern, Bern, Switzerland; Max Planck Research Group at the Malopolska Centre of Biotechnology, Jagiellonian University, Krakow, Poland; Department of Microbial Pathogenicity Mechanisms, Leibniz Institute for Natural Product Research and Infection Biology - Hans Knoell Institute, Jena, Germany; Max Planck Research Group for RNA Biology, Max Planck Institute for Molecular Biomedicine, Muenster, Germany; Department of Chemistry, Biochemistry and Pharmaceutical Sciences, University of Bern, Bern, Switzerland; Graduate School for Cellular and Biomedical Sciences, University of Bern, Bern, Switzerland; Multidisciplinary Center for Infectious Diseases, University of Bern, Bern, Switzerland

## Abstract

Fungal pathogens threaten ecosystems and human health. Understanding the molecular basis of their virulence is key to develop new treatment strategies. Here, we characterize NCS2*, a point mutation identified in a clinical baker's yeast isolate. Ncs2 is essential for 2-thiolation of tRNA and the NCS2* mutation leads to increased thiolation at body temperature. NCS2* yeast exhibits enhanced fitness when grown at elevated temperatures or when exposed to oxidative stress, inhibition of nutrient signalling, and cell-wall stress. Importantly, Ncs2* alters the interaction and stability of the thiolase complex likely mediated by nucleotide binding. The absence of 2-thiolation abrogates the *in vivo* virulence of pathogenic baker's yeast in infected mice. Finally, hypomodification triggers changes in colony morphology and hyphae formation in the common commensal pathogen *Candida albicans* resulting in decreased virulence in a human cell culture model. These findings demonstrate that 2-thiolation of tRNA acts as a key mediator of fungal virulence and reveal new mechanistic insights into the function of the highly conserved tRNA-thiolase complex.

## Graphical Abstract

**Graphical Abstract:**
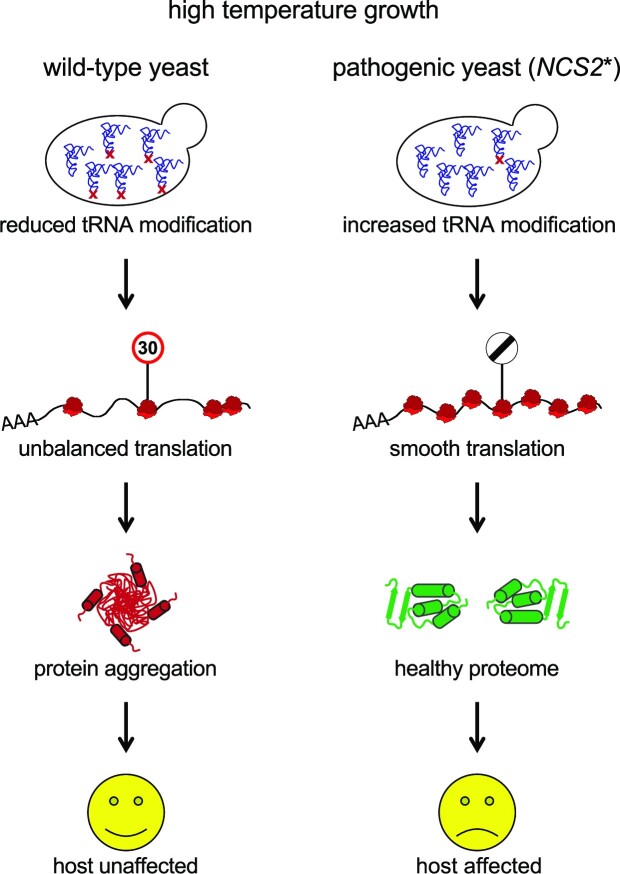

## INTRODUCTION

Fungal pathogens threaten human nutrition and health as well as animal biodiversity (1,2). In general, mammals are resistant to most fungi and only very few species are part of their microbiomes where they usually remain outside epithelial barriers as benign colonizers (3). Some fungal pathogens can cause superficial infections, like *Candida*-associated thrush or dermatophyte-associated skin disorders. However, if a fungus establishes a systemic bloodstream infection, even optimal medical care does not prevent high mortality rates (4). A key factor underlying the basal anti-fungal resistance is the high body temperature of mammals, to which most fungi are sensitive. This has led to a stark interest in identifying genes required for high-temperature growth, since such genes likely facilitate systemic infections (5,6). *Candida albicans* is frequently present in the human gastrointestinal tract as a part of the regular gut microbiome of healthy individuals (3). While it harmlessly inhabits mucosal surfaces, under certain predisposing conditions it can enter the bloodstream and lead to infection of internal organs and trigger septic shock. Indeed, it is one of the most common causes of nosocomial fungemias. *C. albicans* is a polymorphic organism that grows, among others, in a yeast form and in a filamentous form, which are both critical during different stages of infection. The ability of *C. albicans* to trigger the morphological switch as a rapid response to varying growth conditions has been linked to its virulence (7). In contrast, baker's yeast is universally associated with human lifestyle through different fermentation processes and is Generally Recognized As Safe (GRAS) (8). However, if able to grow at high temperatures, *Saccharomyces cerevisiae* can turn into an opportunistic pathogen and infect immunocompromised patients (9). This phenomenon is becoming increasingly relevant in the clinical routine due to rising numbers of patients with an impaired immune system (10).

**Figure 1.**
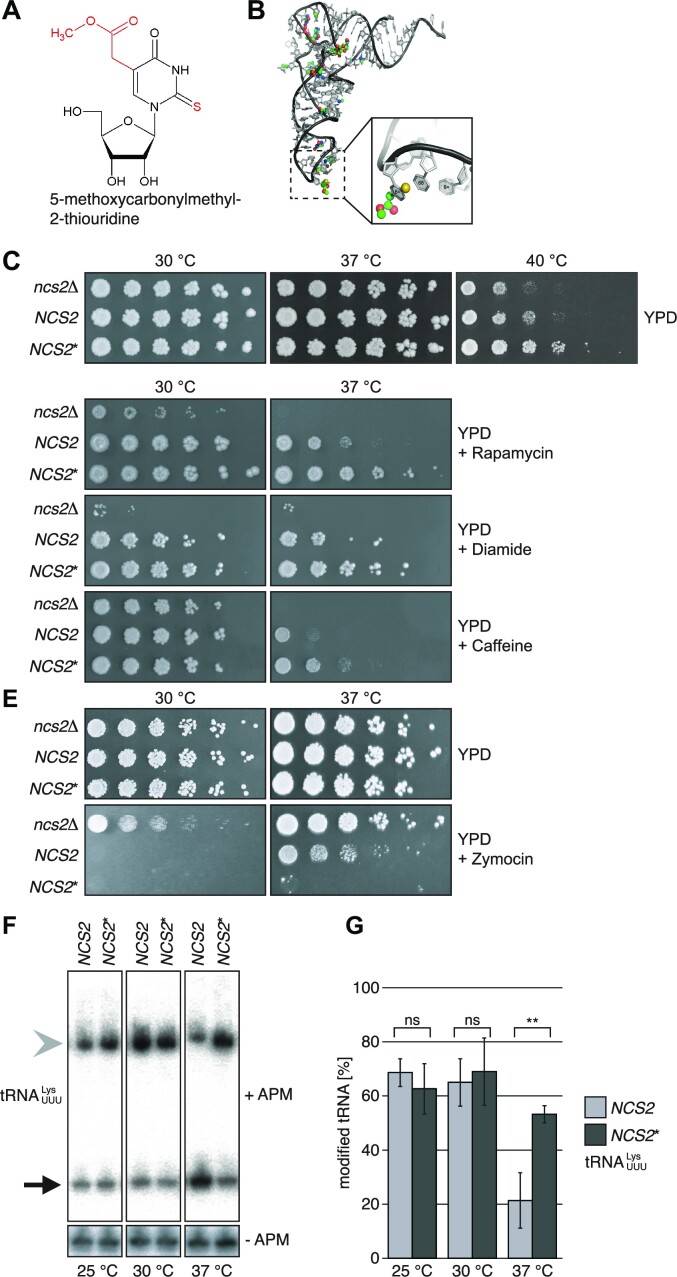
NCS2* affects yeast growth and tRNA 2-thiolation. (**A**) Chemical formula of 5-methoxycarbonylmethyl-2-thiouridine (mcm^5^s^2^U); modifications are indicated in red. (**B**) Structure of human ${\mathrm{tRNA}}_{{\mathrm{UUU}}}^{{\mathrm{Lys}}}.$ Posttranscriptional chemical modifications are indicated as coloured spheres. Inset: mcm^5^s^2^U highlighted by spheres (sulphur: yellow; carbon: green; oxygen: red). (C–E) Spot-dilution assays on YPD using isogenic yeast strains. (**C**) Growth at different temperatures. (**D**) Growth under different chemical stress conditions at 30 °C and 37 °C (2.7 nM rapamycin; 1.5 mM diamide; 6 mM caffeine). (**E**) Growth assay on zymocin. (**F**) Northern-blot analysis of tRNA from wild type and NCS2* yeast grown at 25 °C, 30 °C and 37 °C in YPD. The probe targets ${\mathrm{tRNA}}_{{\mathrm{UUU}}}^{{\mathrm{Lys}}}$; the upper gel contains ([*N*-acryloylamino]phenyl)mercuric chloride (APM). (**G**) Quantification of the fraction of s^2^U-labeled tRNA of northern-blot replicates including (F). *NCS2*: 69 % ± 5 % versus NCS2*: 63 % ± 9 % at 25 °C, *P* = 0.385; *NCS2*: 65 % ± 9 % versus NCS2*: 69 % ± 12 % at 30 °C, *P* = 0.671; *NCS2*: 21 % ± 10 % vs. NCS2*: 53 % ± 3 % at 37 °C, *P* = 0.007 (*n* = 3). Data are represented as mean ± SEM. ** = *P* ≤ 0.01, ns = *P* > 0.05 (two-sided Student's *t*-test).

Transfer RNAs (tRNAs) are the adaptor molecules that physically link messenger RNA (mRNA) codons to their respective amino acid during ribosomal decoding. Importantly, tRNAs carry a plethora of chemical modifications that affect all aspects of tRNA biology (11). Modifications in the anticodon loop tune codon-anticodon interactions and their absence can perturb mRNA translation by affecting the accuracy, speed, and efficiency of protein synthesis (12,13). Therefore, perturbations in tRNA modifications are linked to a growing list of degenerative and metabolic human diseases as well as cancer (14–16). A highly conserved group of chemical tRNA modifications are those of wobble uridine (U_34_) (Figure 1A and B). Nearly all U_34_ in eukaryotic tRNA are modified by the Urm1- or the Elp pathway (for a recent review, see (17)). The Urm1 pathway consists of four essential core members. Ubiquitin related modifier 1 (Urm1) and its activating enzyme Ubiquitin-activating 4 (Uba4) receive and activate sulphur from the cysteine desulphurase NiFS-like (Nfs1) enzyme (18). The activated sulphur is subsequently transferred to the Needs-Cla4-to-Survive 2/6 (Ncs2/Ncs6) complex. Ncs6 is a Fe/S-cluster-containing enzyme (19,20) that adenylates target tRNAs and performs the final sulphur-transfer reaction to generate 5-methoxycarbonylmethyl-2-thiouridine (mcm^5^s^2^U) (Figure 1A and B) (17). Ncs2, the homologous binding partner of Ncs6 has lost its enzymatic function and likely acts as a structural scaffold or provides target specificity (21). Nevertheless, Ncs2 is essential for 2-thiolation and *ncs2*Δ yeast lacks mcm^5^s^2^U-modified tRNA (21,22). The Elongator protein (Elp) complex catalyses the first steps to synthesize 5-methoxycarbonylmethyluridine (mcm^5^U) and 5-carbamoylmethyluridine (ncm^5^U) on U_34_. The dodecameric Elp complex contains two copies of each six individual protein subunits (Elp1–6), all of which are essential for ncm^5^U/mcm^5^U formation (23,24). While s^2^ is not required for mcm^5^ synthesis, the absence of Elongator function leads to a significant decrease in s^2^ levels in yeast. Remarkably, the lack of mcm^5^s^2^U_34_ induces a codon-specific slowdown of mRNA translation (12,13) and thereby triggers protein-homeostasis defects (13). Furthermore, the absence of U_34_ modifications induces complex phenotypes (25,26,21). Interestingly, a mutation in *NCS2* was reported to underlie the ability of a clinical isolate of *S. cerevisiae* to grow at high temperatures (5). This observation prompted us to ask whether tRNA 2-thiolation directly contributes towards the virulence potential of pathogenic yeasts.

Here, we demonstrate that a single nucleotide polymorphism (SNP) in *NCS2*, found in a patient-derived baker's yeast strain, is sufficient to promote yeast virulence. Specifically, yeast harbouring this H71L mutation (NCS2*) grows at high temperature and is resistant to oxidative stress, inhibition of nutrient signalling, and cell-wall stress in a 2-thiolation-dependent manner. NCS2* is characterized by increased levels of 2-thiolation at body temperature compared to wild type. We find that Ncs6 binds Ncs2* more tightly than Ncs2 and that the thiolase complex remains more stable providing a molecular explanation for the increased activity of the 2-thiolation pathway in NCS2* yeast. Furthermore, we observe lower levels of protein aggregates in NCS2* yeast when challenged with high temperatures compared to the wild type. Finally, we show that in the absence of *NCS2* function *S. cerevisiae* and the common pathogen *C. albicans* are less virulent *in vitro* and *in vivo*. These findings reveal the physiological importance of the highly conserved tRNA modification mcm^5^s^2^U as a key mediator of fungal virulence.

## MATERIALS AND METHODS

### Yeast strains and growth conditions

Experiments were conducted in S288c (BY4741), the obligate diploid yeast strains YJM128, YJM145, YJM223, YJM312 and YJM421 to model pathogenic *S. cerevisiae* strains (9,27) and SN87 (28,29) as the parental strain for *Candida albicans*. To generate isogenic *S. cerevisiae* strains, the *NCS2* gene was replaced by a G418 selection cassette. Subsequently, the different alleles of *NSC2* were reintroduced using the pGR-plasmid system (Gwenael Rabut, unpublished) replacing the G418 cassette by the *HIS3* selection marker. To create the isogenic *C. albicans* strain SN87HL, SN87 was transformed with the CIp10HL plasmid to introduce the same *HIS1* and *LEU2* selection markers as in the mutants. Yeasts were grown in YPD (Formedium) at 30 °C, 200 rpm unless stated otherwise. Yeast overnight cultures were diluted to OD_600_ 0.2, grown at the indicated temperatures to OD_600_ 0.8–1.0 and harvested by centrifugation for 3 min at 5000 g unless stated otherwise. Yeast pellets were snap frozen in liquid nitrogen and stored at −80 °C. Pellets of *C. albicans* were washed once with ethanol to kill the cells before freezing. For some experiments pre-cultures were grown at 37 °C to ensure a full reduction of 2-thiolation levels in wild-type yeast. All yeast strains are listed in Supplementary Table S2.

### Spot-dilution assays

Overnight cultures in YPD were diluted to OD_600_ 0.75 in sterile water. A serial dilution of 1:5 was prepared in 96-well plates and spotted onto YPD agar plates adding drugs as indicated. The plates were briefly dried at room temperature and subsequently incubated for 2–3 days at the designated temperature. To investigate the ability of baker's yeast to invade agar, cultures were spotted on YPD plates. Following incubation for 2 days at the appropriate temperature, yeasts were washed off with sterile water and the plates were dried and incubated for one additional day at 30 °C.

### Isolation and purification of tRNA and total RNA

Bulk tRNA was isolated as previously described (30). To isolate total RNA, smaller culture volumes (2–4 ml) were used. Frozen cell pellets were resuspended in 500 μl of 0.9 % NaCl, adding 500 μl acidic phenol and 75 μl 1-bromo-3-chloropropane (BCP). One vortexing step was carried out for 10 min and the aqueous phase was transferred to tubes containing 500 μl acidic phenol and 50 μl BCP. RNA was precipitated overnight in 2 ml ethanol and pellets were washed with 80 % ethanol. The dried RNA was resuspended in 10 μl RNase-free water.

### tRNA 2-thiolation analysis

0.4 μg of bulk tRNA or 1 μg of total RNA per sample were heated for 5 min at 80 °C and applied onto denaturing polyacrylamide gels (8 % polyacrylamide (19:1), 7 M urea, with or without 0.05 % ([*N*-acryloylamino]phenyl)mercuric chloride (APM) in 0.5 × TBE) (31). RNA was transferred onto Immobilon NY^+^ membranes (Millipore) by semi-dry transfer with 0.5× TBE. Northern blot was performed essentially as described using specific DNA-oligo probes (21). Signals were collected and analysed using a Fuji FLA 7000 phosphoimager (Fujifilm). Digital image analysis was performed using Fiji-ImageJ2 (The Fiji Project).

### Isolation of endogenous protein aggregates from yeast

To isolate the endogenous protein aggregates, overnight cultures grown at the indicated temperatures were diluted to OD_600_ 0.05 in 100 ml prewarmed YPD and harvested by filtration, at OD_600_ 0.5. The yeast pellet was snap frozen in liquid nitrogen and stored at −80 °C. Aggregate isolation was performed essentially as described (32). Lysates were prepared by resuspending frozen yeast pellets in 1 ml resuspension buffer (20 mM NaPi pH 6.8, 1 mM EDTA, 10 mM DTT, 0.1 % Tween 20) that contained a cocktail of protease inhibitors (0.5 mM AEBSF, 10 μg/ml aprotinin, 0.5 mg/ml benzamidine, 20 μM leupeptin, 5 μM pepstatin A). 60 U of zymolyase was added and cells were incubated for 20 min at 22 °C, 1400 rpm in a thermo-shaker (Eppendorf) and afterwards chilled on ice. Lysates were transferred to a cold 50 ml reaction tube containing 1 ml of resuspension buffer and sonicated with 8 pulses at level 4, duty cycle 50 % with a tip sonicator (Branson) and chilled on ice. Centrifugation for 20 min at 200 g, 4 °C was carried out in 2 ml reaction tubes. Protein concentration of the supernatant was determined by Bradford analysis. Subsequently, total protein of all samples was adjusted with buffer AIB to equal amounts (1–2.5 mg per sample). 50 μl of this solution was kept as total-protein control. The remaining lysates were centrifuged for 20 min at 16 000 g, 4 °C. The supernatant was discarded, and the pellets were washed in wash buffer 1 (20 mM NaPi pH 6.8, 2 % NP-40 cOmplete protease inhibitor cocktail (Roche)), vortexing the samples till the pellets were dislodged and sonication with 6 pulses at level 4, duty cycle 50 % followed by centrifugation as before. This step was repeated once. The supernatant was discarded and 2 ml of wash buffer 2 (20 mM NaPi pH 6.8, cOmplete protease inhibitor cocktail (Roche)) was added, the samples were vortexed and sonicated with 4 pulses at level 2, duty cycle 65 %, transferred to 2 ml reaction tubes and centrifuged for 20 min at 16 000 g, 4 °C. The supernatant was discarded and all remaining liquid carefully aspirated. Protein-aggregate pellets were resuspended in 100 μl 4× sample buffer containing 8 M urea, to 50 μl of the total protein control samples 25 μl of the same buffer were added and all samples boiled for 10 min at 95 °C and stored at −20 °C. 10 μl of each sample was applied to 12 % polyacrylamide gels and stained with Colloidal Blue (Invitrogen).

### Pulse-chase protein-turnover experiments

Yeast overnight cultures were diluted to OD_600_ 0.2 in YPD and incubated at 30 °C or 37 °C. After 3.5 h, cycloheximide was added to a final concentration of 200 μg/ml. Samples were harvested by centrifugation, cell pellets were resuspended in PBS and adjusted to the 9 OD_600_ units. Proteins were isolated as described previously (33), applied to 10 % polyacrylamide gels and analysed by Western blot.

### Western blot analysis

Following gel electrophoresis, proteins were transferred onto PVDF membranes (Immobilon-P, Millipore). Membranes were blocked in 5 % dry-milk and the primary antibodies were applied at 4 °C overnight. Subsequently, the membrane was washed three times in TBST and incubated with the secondary antibody for 1 h at room temperature. After three washes in TBST, the membrane was incubated with ECL Western Blotting Detection Reagent (GE Healthcare) and visualized on Fuji medical X-Ray film (FujiFilm). Films were digitalized by a scanner (Canon MP 560) and image analysis was performed using Fiji-ImageJ2 (The Fiji Project). Primary antibodies: rabbit anti-TAP (1:10 000; Thermo Fisher, CAB1001); mouse anti-HA (1:8000; Covance MMS-101R). Secondary Antibodies: donkey anti-rabbit HRP (1:20 000; Biorad, 644005); goat anti-mouse HRP (1:10 000; Biorad, 0300-0108P).

### Yeast two-hybrid assays

Y1026 yeast transformed with the yeast-two-hybrid plasmids was grown in SRaf-HT medium overnight. On the next morning, the cultures were diluted in SRaf-HT to OD_600_ 0.2 and incubated at 30 °C to an OD_600_ 0.55–0.65). Subsequently, galactose was added to a final concentration of 2 % and incubated for 1.5 h. The OD_600_ was measured and used for calculating Miller Units (MU). All subsequent steps were carried out on ice. 1 ml of each sample was centrifuged for 3 min at full speed. The pellets were washed with 1 ml resuspension buffer (60 mM Na_2_HPO_4_, 10 mM KCl, 40 mM NaH_2_PO_4_, 1 mM MgSO_4_) and centrifuged as above. The supernatant was discarded, and pellets were resuspended in 150 μl of fresh resuspension buffer containing 0.27 % β-mercaptoethanol. After adding 50 μl BCP and 20 μl of 0.1 % SDS samples were mixed for 10–15 s using a vortex. The reaction was started by adding 700 μl of prewarmed ONPG. When the reaction mix turned yellow, the reaction was stopped by adding 500 μl fresh 1 M Na_2_CO_3_. Samples were centrifuged for 3 min at full speed and OD_420_ of the supernatant was determined using a Synergy Mx plate reader (BioTek). Miller Units were calculated as MU = (OD_420_*1000)/(OD_600_*x min*1 ml), where *x* is the time (in min) that passed between adding ONPG and Na_2_CO_3_.

### Quantification of cytotoxicity (LDH assays)

Confluent monolayers of A498 or C2BBe1 cells in 96-well plates were infected for 24 h with *C. albicans* (MOI = 1) from an overnight culture, incubated at 37 °C in YPD. Cytotoxicity was determined as reported before by a colorimetric assay that measures lactate dehydrogenase (LDH) release using a cytotoxicity detection kit (Roche) according to the manufacturer's instructions (34). Damage by mutant strains was calculated (after subtraction of spontaneous release in all samples) as percent of LDH release caused by the isogenic wild-type strain SN87HL.

### *In vivo* infection experiments

For *in vivo* infection experiments strains carrying two copies of *NCS2*, NCS2*, or *ncs2*Δ were derived from YJM128. 3⋅10^7^ yeast cells of each strain were resuspended in 200 μl sterile PBS and injected into the tail vein of 3–4-week-old female NOD.CB17/scid mice (*n* = 18 animals per group). Mice were allowed to eat and drink *ad libitum* and were monitored three times daily. The experiment was terminated either when mice showed signs of clear distress or when the endpoint was reached after 5 days. The experiments were performed in a blinded manner. Survival curves were analysed using Prism (GraphPad). The use of mice followed the ethical guidelines of the European Laboratory Animal Science Associations (FELASA). All experiments received the ethical approval issued by the Landesamt für Natur, Umwelt und Verbraucherschutz (LANUV) of the state of North Rhine-Westphalia, Germany (Permit 84–02.04.2013.A018).

### Ribosome profiling

Ribosome profiling libraries were prepared as described previously (35–37). Briefly, wild-type and *ncs2*Δ/Δ *C. albicans* yeast strains were grown over night, re-diluted and grown in YPD to OD_600_ 0.4. Cells were harvested by vacuum filtration using a 0.45 μm nitrocellulose filter (GE Healthcare) and immediately flash frozen in liquid nitrogen. The cells were lysed using a Freezer-Mill (SPEX SamplePrep) with the settings: 2 cycles of 2 min precooling, 2 min at 5 CPS and 1 min intermittent cooling. Lysates were thawed in lysis buffer (20 mM Tris–HCl pH 7.4, 5 mM MgCl_2_, 100 mM NaCl, 1 % Triton, 2 mM DTT, 100 μg/ml CHX and clarified by two rounds of centrifugation (3 min; 4 °C; 3000 g and 5 min, 4 °C and 10 000 g). 10 *A*_260_ units of cleared lysates were digested with 250 U Ambion RNase I (ThermoFisher) for 1 h at 22 °C and 1400 rpm agitation. The reaction was stopped by adding 15 μl SuperaseIn (ThermoFisher). To isolate monosomes, the digested RNA was loaded on a 10–50 % sucrose gradient and spun in an ultracentrifuge (SW41 TI rotor; 3 h; 4 °C; 35 000 rpm). Fractions containing monosomes were collected using a piston gradient fractionator (Biocomp) and RNA was extracted with acidic phenol and BCP. Purified RNA was separated on a 15 % PAA gel and ribosome protected fragments (27–29 nt) were excised and precipitated using 0.3 M NaOAc. RPF library preparation was carried out as described before using a 3′-adapter carrying 4 randomized positions at the 5′ end (36,37). Sequencing was performed on an Illumina HiScanSQ instrument.

### Ribosome profiling analysis

Ribosome profiling reads were processed as described (37): Adapter sequences were clipped and the 4 randomized nucleotides trimmed using the FASTX-Toolkit (http://hannonlab.cshl.edu/fastx_toolkit, June 2017), version 0.0.13. The processed reads were mapped to ORFs (cgdGene) using bowtie. Reference ORFs (Allele A, C_albicans_SC5314_version_A22) were extended by 18 nt into the UTRs. A-site codons - excluding footprints that mapped to the first and last 15 codons of a transcript - were mapped according to the frame of the 5′ end of footprints and an appropriate offset was defined (13). For differential gene-expression analysis, gene count tables were generated and the analysis was performed using DESeq2 (38). For altered transcripts, the padj a-threshold was set to 0.05. To identify unaltered transcripts, the althypothesis function was set to ‘lessAbs’. Gene ontology (GO) enrichment analysis used TopGO. Redundant GO-terms were removed using Revigo (39).

### Sequence analysis

Sequences (NP_014280.3 (*S. cerevisiae*), NP_595698.1 (*S. pombe*), NP_001260585.1 (*D. melanogaster*), NP_001012777.1 (*H. sapiens*), Q6DC53.2 (*D. rerio*), KHC70741.1 (*C. albicans*), and NP_505729.1 (*C. elegans*) were analysed using T-Coffee (tcoffee.crg.cat) using the standard MSA settings (40,41). The colour of the amino acids indicates the likelihood that the residues are correctly aligned (blue: 0, dark red: 9). Values >5 (yellow, orange, and red) are likely correctly aligned. The alignment was manually edited to match the format size and minor alignment changes were introduced in low-quality regions of the alignment.

### Structural models

We have built structural models of the Ncs2/6 heterodimer using the AlphaFold 2 Protein Structure Database (42) and the Protein Homology/analogy Recognition Engine (Phyre2) (43). Primary structural features of the complex were obtained from the crystal structures of TtuA (PDB: 3VRH, 5ZTB), an archaeal structural homolog of Ncs6 in *T. thermophilus* (44,45). The tRNA positioning was inferred from the crystal structure of TilS-tRNA (PDB: 3A2K), a lysidine synthetase from *Geobacillus kaustophilus*, superimposed on the Ncs6 subunit.

### CtNcs2 & CtNcs6 protein sample preparation

The sequences of *C. thermophilum* (Ct) Ncs2 (UniProt ID: G0SFJ9) and CtNcs6 (UniProt ID: G0SAK0) were codon-optimized for expression in *Escherichia coli*, obtained from Genscript, and cloned into pETM30 (providing a TEV cleavable N-terminal 6xHis- and GST- tag) vectors. The proteins were expressed in BL21 (DE3) pRARE in LB at 18 °C using overnight induction with 0.5 M IPTG. Bacterial pellets were resuspended in lysis buffer (50 mM Tris–HCl pH 8, 300 mM NaCl, 0.15 % TX-100, 10 mM MgSO_4_, 1 mM DTT, 10 mg/ml DNase, 1 mg/ml lysozyme, 10 % glycerol and a protease inhibitor cocktail) and lysed to homogeneity using a high-pressure homogenizer Emulsiflex C3 (Avestin). To form the CtNcs2/6 complex, pellets containing each construct were mixed during lysis. The proteins were purified using GSTrap (Cytiva) columns on an ÄKTAstart (Cytiva) under standard conditions. Tags were optionally cleaved with TEV protease and removed in a second GSTrap purification step. Subsequently, the proteins were purified from nucleic acid contaminants using a HiTrap Heparin HP (Cytiva) column. Finally, proteins were purified by size-exclusion chromatography on a HiLoad 26/600 Superdex 200 prep-grade column (Cytiva). Purified proteins were stored at −80 °C in storage buffer (20 mM Tris pH 8, 150 mM NaCl and 1 mM DTT).

### GST-pulldown for ncs2/6 interaction

CtNcs2 or ChtNcs6 proteins were incubated with GST-CtNcs6 and GST-CtNcs2 respectively, in reaction buffer (20 mM Tris pH 8, 150 mM NaCl and 1 mM DTT). The reaction mix was subsequently added to equilibrated Glutathione Sepharose 4B beads (GE Healthcare) and incubated on a rotating wheel for 120 min at 4 °C. After binding, glutathione beads were collected by gentle spinning at 500 g and subsequently washed three times with a 0.05 % (v/v) Tween 20-containing reaction buffer. Bound proteins were denatured at 95 °C in the presence of Laemmli sample buffer and analysed on BoltTM 4–12 % Bis–Tris Plus Gels (Thermo Fisher Scientific). For protein visualization, the gels were stained with Coomassie Brilliant Blue. Inputs were collected after the reaction and before the pull-down and represent 7 % of the pull down.

### Thermal-shift assays

To stabilize CtNcs6, 10 μg of either CtNcs2, CtNcs6 or CtNcs2/6 were suspended in 50 mM HEPES pH 8, 150 mM NaCl, 2 mM DTT. The influence of nucleotides on the thermostability of CtNcs2 and CtNcs2/6 was tested in the storage buffer supplemented with 2 mM MgCl_2_. Each protein was measured in a buffer without nucleotide, with 1 mM ADP, 1 mM ATP or 1 mM AMP-PNP. Samples were gradually heated 4–98 °C for 2 h. The denaturation process was tracked using the hydrophobic fluorescent dye SYPRO Orange (Sigma Aldrich). Fluorescence was measured using a Bio-Rad CFX96 thermocycler. Melting temperatures were calculated from the average of the peaks of the first derivative from at least three technical replicas.

## RESULTS

### NCS2* confers cellular fitness under stress

A screen for mutations that enable clinical isolates of baker's yeast to grow at high temperatures identified a SNP in *NCS2* (A212T), which leads to a histidine-to-leucine substitution (H71L) (5). Since Ncs2 is a component of the tRNA-thiolase complex (17), we sought to investigate the role of this highly conserved tRNA modification in yeast virulence. We used the common laboratory yeast background S288c to generate isogenic strains that only differ by this SNP (further called *NCS2* for the wild-type allele and NCS2* for the H71L mutant; Supplementary Figure S1A). First, we grew the strains on YPD at 30 °C, 37 °C and 40 °C and confirmed that NCS2* yeast grew better than *NCS2* or *ncs2*Δ yeast at 40 °C, while we observed no difference at lower temperatures (Figure 1C). Next, we exposed the strains to conditions that require 2-thiolation. NCS2* grew better than the wild type when challenged with the TOR inhibitors rapamycin and caffeine, the sulfhydryl-oxidizing agent diamide, the cell-wall damaging reagent calcofluor white (CFW) or paromomycin at 37 °C (Figure 1D and Supplementary Figure S1B; Supplementary Table S1). Furthermore, we treated yeast with zymocin, a toxin that specifically cleaves mcm^5^s^2^U-modified tRNA, while hypomodified tRNA is resistant to nucleolytic cleavage (46–48). Strikingly, NCS2* yeast did not grow at 37 °C (Figure 1E) while *NCS2* and *ncs2*Δ yeast grew under these conditions. When performing similar experiments at 30 °C, the difference between *NCS2* and NCS2* was negligible (Figure 1E). Notably, in wild-type yeast, s^2^U levels in tRNA are reduced at elevated temperatures, which is consistent with the observed effect in response to zymocin (30,49–51). Therefore, we compared the levels of tRNA 2-thiolation in *NCS2* and NCS2* yeast at different temperatures by ([*N*-acryloylamino]phenyl)mercuric chloride (APM)-affinity gel electrophoresis and northern blotting (31). 2-thiolation levels were similar in *NCS2* and NCS2* at 25 °C and at 30 °C (Figure 1F, G and Supplementary Figure S1C-S1F). However, at 37 °C 2-thiolation was significantly decreased in *NCS2*, while remaining stable in NCS2* yeast (Figure 1F, G and Supplementary Figure S1C-S1F). Our results show that the NCS2* mutation confers resistance to temperature and chemical stress and that this effect is likely mediated by 2-thiolation. Furthermore, our data confirm that elevated temperatures regulate the activity of the modification pathway consistent with previous findings (30,49–51).

### The cellular effects of NCS2* are mediated through 2-thiolation of tRNA

**Figure 2.**
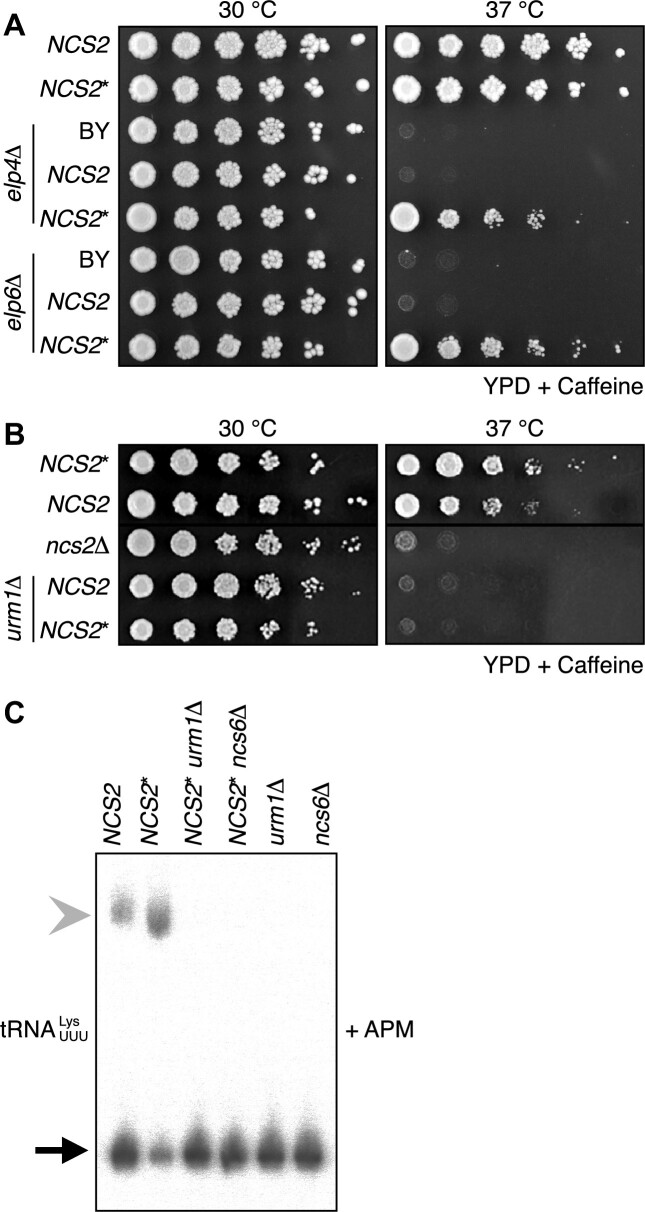
NCS2* modulates tRNA functionality in a thiolation-dependent manner. (A, B) Spot-dilution assay of *NCS2* and NCS2* strains in combination with gene deletions that affect wobble uridine modifications grown on YPD. (**A**) *NCS2* and NCS2* in combination with *elp4*Δ and *elp6*Δ (3 mM caffeine). (**B**) *NCS2* and NCS2* in combination with *urm1*Δ (6 mM caffeine). (**C**) Northern-blot analysis of total RNA from different yeast strains grown at 37 °C in YPD. The probe is against ${\mathrm{tRNA}}_{{\mathrm{UUU}}}^{{\mathrm{Lys}}}$. The gel contains ([*N*-acryloylamino]phenyl)mercuric chloride (APM).

Since Ncs2 is required for 2-thiolation of U_34_, we asked whether NCS2* confers cellular fitness by affecting tRNA functionality. Therefore, we deleted genes encoding for members of the Elongator complex (*ELP4* and *ELP6*) and the Urm1 pathway (*URM1* and *NCS6*) in *NCS2* or NCS2* yeast. In the absence of *ELP4* or *ELP6*, xm^5^U_34_ modifications are not formed. Therefore, s^2^ levels are reduced (17,52). Interestingly, the stress sensitivity of *elp4*Δ and *elp6*Δ yeast was rescued in NCS2* but not in *NCS2* yeast (Figure 2A and Supplementary Figure S2A), strengthening a direct link of the phenotype to s^2^U levels. Next, we deleted *URM1* or *NCS6*, which are essential for 2-thiolation and found that the NCS2*urm1Δ and NCS2*ncs6Δ yeasts were indistinguishable from *NCS2urm1*Δ and *NCS2ncs6*Δ yeast under stress at 37 °C, thus, demonstrating that NCS2* exerts its function through 2-thiolation (Figure 2B and Supplementary Figure S2B). Finally, tRNA is not 2-thiolated in NCS2*urm1Δ or NCS2*ncs6Δ yeast (Figure 2C), showing that Ncs2* does not bypass the requirement of the Urm1 pathway. Overall, these findings establish that NCS2* enhances cellular fitness through 2-thiolation of U_34_.

### The NCS2* mutation is specific

**Figure 3.**
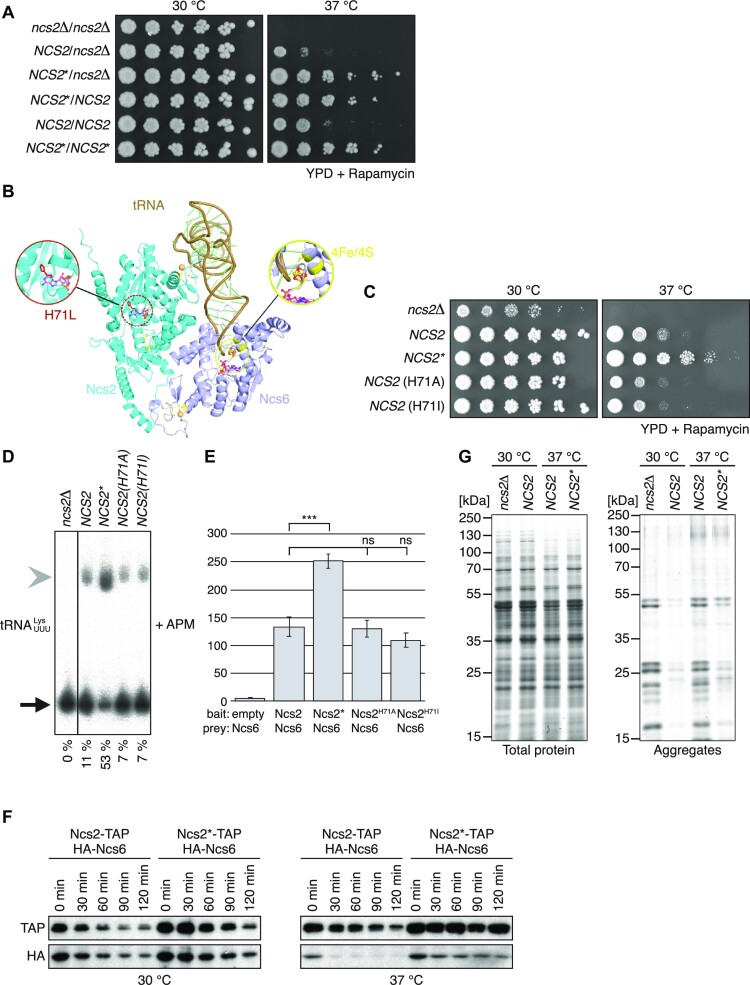
Mechanism of NCS2* function. (**A**) Spot-dilution assay of diploid yeast strains containing combinations of *NCS2* alleles grown on YPD (2.7 nM rapamycin). (**B**) Phyre-based structural model of the Ncs2/6 complex with tRNA in cartoon representation with close-up views of the L71 residue (left) and the catalytic site of Ncs6 (right). Putative binding sites of ATP and the coordination of the iron/sulphur cluster are indicated with ball-and-stick models. Zinc atoms (light brown) positioned in the N- and C- terminal domains of both Ncs2 and Ncs6 are enlarged for visibility (Ncs2: cyan, Ncs6: purple, tRNA: brown). (**C**) Spot-dilution assay of different *NCS2* alleles grown on YPD (2.7 nM rapamycin). (**D**) Northern-blot analysis of total RNA from strains expressing different *NCS2* alleles grown at 37 °C in YPD. The probe targets ${\mathrm{tRNA}}_{{\mathrm{UUU}}}^{{\mathrm{Lys}}}$. The gel contains ([*N*-acryloylamino]phenyl)mercuric chloride (APM). The fraction of s^2^U-labeled tRNA is indicated below. (**E**) Yeast-two-hybrid assay of Ncs6 with Ncs2 variants. Ncs2: 133.5 ± 17.8 Miller units [MU], Ncs2*: 251.8 ± 12.7 MU, Ncs2^H71A^: 130.2 ± 15.1 MU, Ncs2^H71I^: 109.2 ± 12.8 MU. Ncs2/Ncs6 vs. Ncs2*/Ncs6: *P* = 0.0007; Ncs2/Ncs6 vs. Ncs2^H71A^/Ncs6: *P* = 0.814; Ncs2/Ncs6 vs. Ncs2^H71I^/Ncs6: *P* = 0.127 (*n* = 3 independent yeast-two hybrid assays for each construct). Data are represented as mean ± SEM. *** = *P* ≤ 0.001, ns = *P* > 0.05 (Two-sided Student's *t*-test). (**F**) Western blot analysis of yeast strains expressing Ncs6-HA and Ncs2-TAP or Ncs2*-TAP, respectively. Translation was blocked by adding 200 μg/ml cycloheximide to the culture at timepoint 0. Samples were taken every 30 min. (**G**) Insoluble protein aggregates were isolated from *ncs2*Δ, *NCS2* or NCS2* yeast grown in YPD at 30 °C or 37 °C, respectively. Proteins were separated by SDS-PAGE and subsequently visualized by Colloid Coomassie staining (left: total extracts; right: aggregates).

*S. cerevisiae* naturally and predominantly exists in a diploid form. Therefore, we asked whether a single allele of NCS2* is sufficient to confer a fitness advantage in S288c. To address this question, we generated a series of isogenic diploid strains with all possible combinations of the NCS2*, *NCS2* and *ncs2*Δ alleles. While *ncs2*Δ/*ncs2*Δ yeast was sensitive to stress when grown at 37 °C, a single copy of the *NCS2* allele partially rescued growth at high temperatures (Figure 3A). However, a single copy of the NCS2* allele resulted in a stronger rescue when combined with either *ncs2Δ* or *NCS2*, recapitulating the high-temperature growth phenotype (Figure 3A). Remarkably, this shows that NCS2* is dominant over the wild-type allele and suggests that a single copy of NCS2* is sufficient to confer a growth advantage.

To understand the molecular mechanism by which Ncs2* exerts its fitness advantage we analysed its sequence. Ncs2 does not contain known functional domains. However, the H71L substitution occurs within a short region that is relatively well conserved in Ncs2 (Supplementary Figure S3A; black box). We generated three-dimensional models of Ncs2* in complex with Ncs6 and a tRNA. The first model was based on the structures of TtuA from *Thermus thermophilus*—a sulphurtransferase that is homologous to Ncs2/Ncs6—and tRNA-bound TilS from *Geobacillus kaustophilus* (Figure 3B), and the second very consistent model is based on an AlphaFold 2 prediction (Supplementary Figure S3B) (44,45). The anticodon loop of the tRNA fits into the catalytic pocket of Ncs6 in the vicinity of its ATPase domain and its 4Fe/4S-iron-sulfur cluster. The C-terminus of Ncs2 contacts the core of the tRNA, thereby likely stabilizing the tRNA-thiolase complex in line with its potential role as a specificity factor (21). The H71L substitution is located ∼25 Å away from the Ncs2-tRNA interface in a region that is homologous to the ATPase domain of Ncs6. We next asked whether the high-temperature growth phenotype of NCS2* yeast was specific to the H71L substitution or whether any other amino acid substitution was sufficient to recapitulate the phenotype. Therefore, we replaced H71 with alanine (H71A) or isoleucine (H71I). However, yeast carrying these substitutions did not show a fitness advantage (Figure 3C). Furthermore, 2-thiolation levels were reduced in both mutants at 37 °C (Figure 3D). Our results show that the enhanced fitness is specific to the H71L substitution and that it occurs by increasing 2-thiolation levels at high temperatures.

### The H71L substitution enhances thiolase-complex stability

Since Ncs2 forms a heterodimer with Ncs6 in different organisms (53,21), we asked whether the formation of the thiolase complex underlies the fitness advantage of Ncs2*. Therefore, we probed the interaction between Ncs6 and the Ncs2 variants by yeast-two-hybrid (Figure 3E). Strikingly, the interaction of Ncs6 with Ncs2* was approximately 2-fold higher than between Ncs6 and Ncs2, while the interactions with Ncs2^H71A^ or Ncs2^H71I^ were similar or slightly weaker than with the wild-type protein (Figure 3E). This led us to hypothesize that the stronger interaction between Ncs6 and Ncs2* stabilizes the activity of the thiolase complex. We tested this by monitoring the turnover of Ncs2, Ncs2*, and Ncs6 *in vivo* after blocking protein synthesis with cycloheximide. Ncs2 and Ncs2* showed roughly similar decay rates at 30 °C or 37 °C (Figure 3F). However, while Ncs6 decayed quickly at 37 °C in *NCS2* yeast, it was more stable in NCS2* yeast (Figure 3F). This shows that stabilization of Ncs6 at high temperatures results in an increase of functional Ncs6/Ncs2*, thus leading to higher tRNA 2-thiolation levels in NCS2* yeast during high-temperature growth. To further analyse the interaction of Ncs2 and Ncs6 we performed *in vitro* experiments. Ncs6 contains an oxygen-sensitive Fe/S cluster (19,20,54). This prevented us from reconstituting the baker's yeast thiolase complex *in vitro*. However, we succeeded in expressing recombinant Ncs2 and Ncs6 from *Chaetomium thermophilum*. As expected, both proteins form a complex (Supplementary Figure S3C) (21,53). To determine the stability of the complex and its individual subunits, we used thermal shift assays. We found that the Ncs2/Ncs6 complex is much more stable than Ncs2 or Ncs6 alone (Supplementary Figure S3D). Even though residues critical for an enzymatic function have been mutated (21) it is possible that Ncs2 binds ATP and that this stabilises the protein. Importantly, we found that the stability of Ncs2 increases significantly, when it is bound to ATP, ADP or the non-hydrolysable AMP-PNP (*t*-test < 0.001, *n* = 3–4; Supplementary Figure S3E). Binding to these three nucleotides likewise enhances the stability of the Ncs2/6 complex (*t*-test < 0.001, *n* = 3–4; Supplementary Figure S3F). These data support the hypothesis that the H71L substitution stabilizes the thiolase complex likely by modulating nucleotide binding of Ncs2.

### NCS2* affects protein homeostasis at high temperatures

Codon-specific translation rates are decreased in the absence of U_34_ modifications (12,13), thereby triggering protein-homeostasis defects (13). Therefore, we hypothesized that higher levels of 2-thiolated tRNA leads to decreased protein aggregation in NCS2* cells. To test this, we grew *ncs2*Δ, *NCS2*,\ and NCS2* yeast at 30 °C and 37 °C and monitored the presence of protein aggregates (32,13). Indeed, *ncs2*Δ cells, but not wild-type cells contained protein aggregates at 30 °C (Figure 3G). When grown at 37 °C, both *NCS2* and NCS2* cells contained aggregates, but the amount of aggregated protein was significantly lower in NCS2* yeast (Figure 3G). The accumulation of cytoplasmic protein aggregates constitutes a significant fitness cost and likely explains why NCS2* yeast cope better with stressful environments.

### NCS2* leads to increased virulence *in vivo*

**Figure 4.**
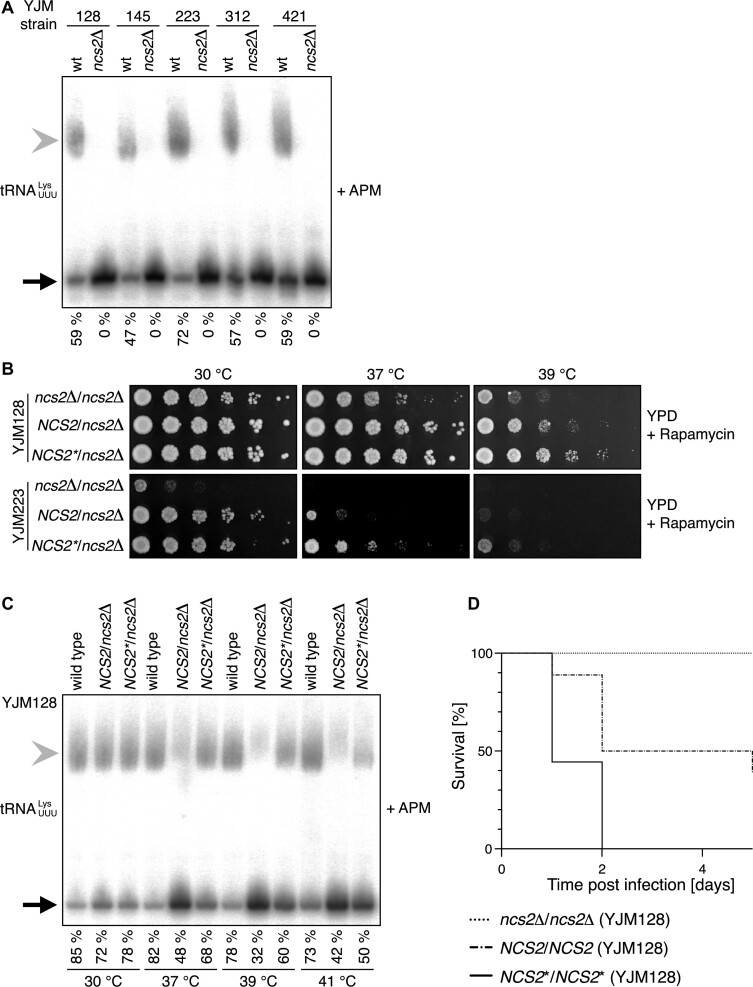
*NCS2* modulates virulence in pathogenic baker's yeast. (**A**) Northern-blot analysis of total RNA from wild type and *ncs2*Δ yeast derived from clinical isolates grown at 30 °C in YPD. The probe is against ${\mathrm{tRNA}}_{{\mathrm{UUU}}}^{{\mathrm{Lys}}}$. The gel contains ([*N*-acryloylamino]phenyl)mercuric chloride (APM). The fraction of s^2^U-labeled tRNA is indicated below. (**B**) Spot-dilution assay of *NCS2* alleles generated in the clinical isolates YJM128 and YJM223 grown on YPD (YJM128: 5 nM rapamycin; YJM223: 4 nM rapamycin). (**C**) Northern-blot analysis of total RNA from YJM128-derived strains shown in (B) grown at different temperatures in YPD. The probe is against ${\mathrm{tRNA}}_{{\mathrm{UUU}}}^{{\mathrm{Lys}}}$. The gel contains APM. The fraction of s^2^U-labeled tRNA is indicated below. (**D**) Survival curves of mice injected with *NCS2*/*NCS2*, NCS2*/NCS2* or *ncs2*Δ/*ncs2*Δ yeast; *P* < 0.0001; Mantel-Cox Log-rank test (*n* = 18 animals per group). The yeast strains used for *in vivo* infection experiments were derivatives of YJM128.

NCS2* was isolated from a clinical isolate of baker's yeast. To directly link tRNA 2-thiolation to yeast pathogenicity, we analysed its role in clinical *S. cerevisiae* isolates (9,27,55). These pathogenic strains differ from laboratory strains in their repertoire of SNPs and are generally diploid. Therefore, we deleted both alleles of *NCS2* in several clinical isolates. As expected, tRNA was not thiolated in these knockouts (Figure 4A). Interestingly, 2-thiolation levels differ between wild-type isolates while most of them were temperature sensitive (Supplementary Figure S4A). We selected YJM128 as an example of a virulent strain and YJM223 as an example of a strain with intermediate virulence (9,27,55). Next, we reintroduced *NCS2* or NCS2* into the corresponding deletion strains. Importantly, both strain backgrounds reacted to stress like the laboratory yeast, showing that the results obtained in S288c are a good proxy for pathogenic baker's yeast (Figure 4B). We subsequently analysed whether 2-thiolation levels differ between *NCS2* and NCS2* at high temperatures in these strain backgrounds and found this to be the case (Figure 4C and Supplementary Figure S4B). However, likely due to the composition of SNP in these pathogenic isolates, a single allele of NCS2* was not sufficient to restore wild-type s^2^U levels, suggesting that the dominant effect of NCS2* that we observed for in S288c requires genetic interactors beyond the H71L substitution. Finally, we tested whether the add-back strains can invade agar. In the YJM128 background, we found improved attachment to the substrate in NCS2* yeast, while none of the derivatives of YJM223 was able to invade agar (Supplementary Figure S4C).

We next determined whether increased 2-thiolation levels in NCS2* yeast confer a fitness advantage when exposed to the immune system of a human host. Thus, we incubated the yeast cells with blood from healthy human donors and monitored the efficiency of the immune cells to kill the yeast. We observed efficient killing in all cases, confirming that the intact human immune system can defend the host efficiently against baker's yeast (data not shown). Therefore, we applied an *in vivo* infection model that mimics an immunocompromised host. We injected yeast into the tail vein of 3–4-week-old NOD.CB17/scid mice and monitored survival for 5 days (56). Strikingly, all mice were resistant to infections by *ncs2*Δ cells (Figure 4D). Furthermore, the mice were more severely affected by NCS2* yeast compared to *NCS2* yeast consistent with an increased fitness of the mutant (Figure 4D). Collectively, these experiments demonstrate that 2-thiolation facilitates virulence when baker's yeast infects a susceptible mammalian host.

### 2-thiolation mediates virulence in *C*.*albicans*

**Figure 5.**
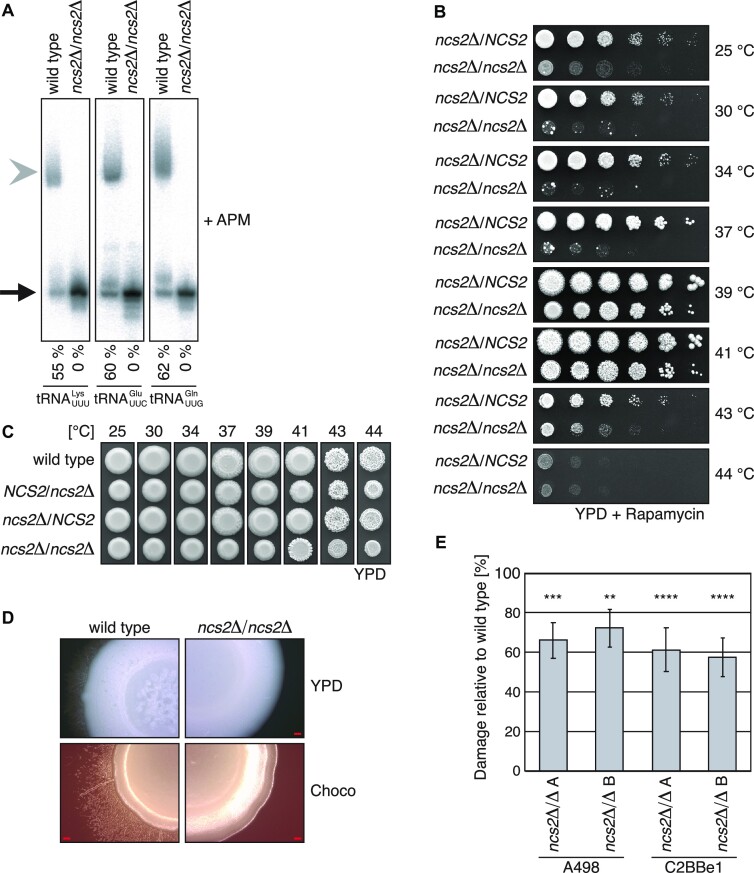
*NCS2* in *Candida albicans*. (**A**) Northern-blot analysis of total RNA from wild type and *ncs2*Δ/Δ *C. albicans* grown in YPD at 30 °C. The probes are against ${\mathrm{tRNA}}_{{\mathrm{UUG}}}^{{\mathrm{Gln}}}$, ${\mathrm{tRNA}}_{{\mathrm{UUC}}}^{{\mathrm{Glu}}}$ and ${\mathrm{tRNA}}_{{\mathrm{UUU}}}^{{\mathrm{Lys}}}$. The gel contains ([*N*-acryloylamino]phenyl)mercuric chloride (APM). The fraction of s^2^U-labeled tRNA is indicated below. (**B**) Spot-dilution assay of *NCS2*/*ncs2*Δ and *ncs2*Δ/Δ cells grown at different temperatures on YPD (10 nM rapamycin). (**C**) Spot-dilution assay of different *NCS2* alleles grown on YPD at different temperatures. (**D**) Wild type and *ncs2*Δ/Δ *C. albicans* grown at 37 °C using conditions that induce hyphae formation (YPD and choco medium). Scale bar is 500 μm. (**E)** Damage to human kidney (A498) and colon (C2BBe1) cell lines as determined by release of host LDH. Damage caused by the *ncs2*Δ/Δ strains is shown compared to the host cell damage elicited by the isogenic wild-type strain SN87HL (100 %) (*n* = 4 or 5 biological replicates; significance levels compared to the wild type 100 %). ** = *P* ≤ 0.01, *** = *P* ≤ 0.001, **** = *P* ≤ 0.0001 (two-sided Student's *t*-test).

Although *S. cerevisiae* can infect immunosuppressed patients, baker's yeast is Generally Recognized As Safe (GRAS). Therefore, we used *C. albicans* to understand how the absence of 2-thiolated tRNA affects the virulence of a genuine fungal pathogen. First, we grew wild-type *C. albicans* at 25–45 °C, purified tRNA from the cells and assessed its thiolation status. This established that 2-thiolated tRNA is present in *C. albicans* and that 2-thiolation levels remain constant at different temperatures (Supplementary Figure S5A). Next, we identified the *C. albicans* orthologue of *NCS2* (orf19.4399) and knocked out both alleles (28,29). tRNA isolated from *ncs2*Δ/Δ cells are not thiolated, demonstrating the conservation of Ncs2 function (Figure 5A). To test whether the *ncs2*Δ/Δ mutant is sensitive to thiolation-linked stress, we challenged it by rapamycin. The *ncs2*Δ/Δ strain was sensitive to the drug at 25–37 °C, but the effect was less pronounced at higher temperatures (Figure 5B). To distinguish 2-thiolation-specific phenotypes from a general stress response, we exposed the strains to the antifungal drugs fluconazole, nystatin, and natamycin (Supplementary Figure S5B). However, we did not observe a difference in sensitivity towards these drugs, suggesting that the lack of 2-thiolation does not lead to general stress sensitivity. To investigate whether a lack of thiolated tRNA affects high-temperature growth we grew the wild-type, heterozygous, and full deletion strains at 25–44 °C (Figure 5C and Supplementary Figure S5C). At temperatures exceeding 37 °C *ncs2*Δ/Δ colonies appeared smoother than the wild type or heterozygous strains. Colony morphology is a good proxy for the ability of the strains to form hyphae. Hence, we grew the strains under hyphae-inducing conditions. Indeed, the *ncs2*Δ/Δ colonies neither formed radial hyphae nor central wrinkling, while wild-type colonies showed robust hyphae formation on YPD or Choco medium (Figure 5D). Finally, we tested whether 2-thiolation-deficient *C. albicans* is able to damage epithelial cells in a cell-culture model. Importantly, we found that *ncs2*Δ/Δ cells exhibit a striking reduction in their ability to damage kidney (A498) or colon (C2BBe1) cells, indicating that they are less virulent than the wild type (Figure 5E). These findings underscore the importance of tRNA modifications for virulence also in the human pathogen *C. albicans*.

### Thiolation-deficient *C. albicans* shows translation defects

**Figure 6.**
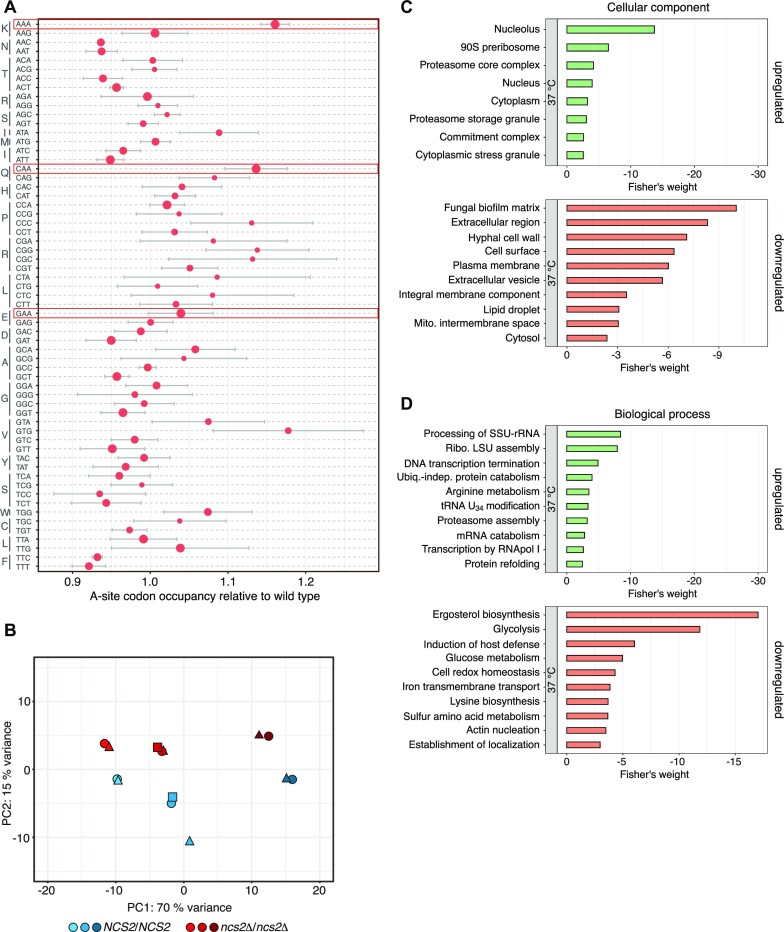
Translation defects in *Candida albicans*. (**A**) Relative A-site codon-occupancy in *ncs2*Δ/Δ cells relative to wild type (mean ± SD; n = 3). Codons cognate for tRNAs with mcm^5^s^2^U_34_ are marked in red. Dot size indicates the relative frequency of each codon in the wild-type A site (the larger the more frequent). (**B**) Principal component analysis (PCA) of gene expression of wild type (blue) and *ncs2*Δ/Δ (red) *C. albicans* grown in YPD at 30 °C (light colour), 37 °C (medium colour), and 42 °C (dark colour). Replicates are indicated as circles, triangles, and squares. (C, D) Gene ontology (GO) terms mis-regulated in *ncs2*Δ/Δ *C. albicans* relative to wild type at 37 °C. (**C**) Cellular component. (**D**) Biological process.

Notably, the presence of 2-thiolation of tRNA has been shown to tune codon-specific translation in different organisms (12,13,57). Therefore, we asked whether a lack of mcm^5^s^2^U_34_ similarly affects translation dynamics in *C. albicans*. To address this question, we performed ribosome profiling of wild-type and *ncs2*Δ/Δ cells. This method generates high-resolution snapshots of translation through the sequencing of ribosome-protected mRNA fragments (58) and has been used to characterize translation defects in tRNA modification mutants (12,13,59). Consistent with baker's yeast, we observed increased codon occupancy for AAA and CAA in the ribosomal A site of *ncs2*Δ/Δ *C. albicans* at 37 °C (Figure 6A) indicating that ribosomes in the mutants require more time to decode codons that depend on hypomodified tRNA (12,13). Next, we performed differential-expression analyses of the ribosome profiling data to identify pathways that differ in activity between the mutants and the wild type. Interestingly, the factor that best explained differences in the gene-expression between the samples was temperature (Figure 6B). Next, we conducted gene ontology (GO) analyses of the genes that are differentially expressed between *ncs2*Δ/Δ cells and wild type. Mutant cells are characterized by changes in their metabolic programs indicated by a reorganization of glucose and amino acid metabolism and the downregulation of genes that encode for proteins that localize to mitochondria (Figure 6C, D and Supplementary Figure S6A-S6D). Furthermore, genes belonging to GO terms that are linked to membrane biology, the hyphal cell wall and biofilm formation were consistently downregulated in the mutant, which is compatible with altered colony morphology and a decrease in hyphal growth. These findings suggest that the observed codon-specific translation defect in *C. albicans* mutants partially blocks the morphological switch to hyphal growth, thereby rendering *C. albicans* less pathogenic. Like pathogenic baker's yeast, *C. albicans* virulence is severely reduced in the absence of mcm^5^s^2^U.

## DISCUSSION

Using a model of pathogenic baker's yeast and *C. albicans*, we demonstrate that 2-thiolation is a key mediator of virulence. Strains that lack mcm^5^s^2^U exhibit decreased fitness at high temperature and fail to infect mice and to damage human cells (Figures 4D and 5E). To our knowledge, this is the first time that a similar phenotype has been shown *in vivo* for fungal pathogens.

NCS2* is a SNP that leads to the substitution of histidine by leucine (H71L) in a conserved region of Ncs2 (Supplementary Figure S3A) (5). The gain of function that is introduced by H71L cannot be explained by a loss of the positive charge, since mutations to alanine (H71A) and isoleucine (H71I) do not mimic the phenotype of H71L (Figure 3C and D). Our structural model of Ncs2 suggests that H71L is located in a putative ATP binding pocket (Figure 3B and Supplementary Figure S3B). Since Ncs2 has mutated several key residues during evolution it is unlikely to hydrolyse ATP (21). However, ATP binding may stabilize Ncs2* compared to Ncs2. This attractive model is in line with our observation that nucleotide binding increases the stability of Ncs2 and the thiolase complex from *C. thermophilum* (Supplementary Figure S3E and F). Notably, the difference between Ncs2 and Ncs2* is a new example that heritable traits giving rise to complex phenotypes like high-temperature growth and virulence can be generated by a single point mutation (60,61). Traits that generate a fitness advantage are likely to become fixed in a population. We found that NCS2* confers a fitness advantage under a range of stress conditions including starvation, oxidative stress, and mistranslation (Figure 1D and Supplementary Figure S1B). Nevertheless, the *NCS2* allele is frequently found in wild yeast isolates. If carrying NCS2* were always advantageous, we would expect to observe NCS2* in all yeast strains. This suggests that NCS2* does not always provide a fitness advantage or even confers a negative effect under specific growth conditions. However, at high temperatures NCS2* becomes advantageous as indicated by its independent isolation in evolution experiments performed at high temperatures (6).

Defects in the Urm1 pathway likewise reduce the ability for high-temperature growth in archaea, yeasts, worms, rice, and bacteria (25,53,62,63), a phenotype that is further enhanced by specific stress conditions. This underscores the physiological importance of mcm^5^s^2^U, consistent with findings that wobble uridine modifications are part of the core cellular translation machinery (64,65). Interestingly, a decrease of 2-thiolation levels at high temperatures is not observed in all organisms. While *Saccharomyces bayanus* and *japonica* rice show low levels of mcm^5^s^2^U similar to baker's yeast, this is not the case in *C. albicans*, *C. glabrata* or *indica* rice (Supplementary Figure S5A) (30,63). This difference might stem from variations in the metabolic wiring of the different species or the enzymatic properties of Urm1-pathway components. In baker's yeast, Urm1 pathway members are less abundant at high temperatures (50,51), likely because of their decreased stability (30,50,51). We show that Ncs6 levels depend on the stability of Ncs2 (Figure 3F). However, it is unclear whether this effect similarly regulates the abundance of proteins like Uba4.

The 2-thiolation machinery is integrated into cellular sulphur fluxes. Interestingly, these findings support a model where mcm^5^s^2^U levels are used by the cells to regulate global translation (66,67). Linking 2-thiolation levels to metabolic states is intriguing, since this mechanism can explain how tRNA modifications regulate global translation in response to the metabolic requirements of the cell (68–70). Nevertheless, the regulation of mRNA translation by tRNA modifications acts slowly due to the high stability of tRNA and the absence of demodifying enzymes for most tRNA modifications. Likewise, 2-thiolation levels respond to high temperatures within several hours in baker's yeast (30) most likely due to decreased enzyme activity at high temperatures. The fact that *Candida* strains and baker's yeast respond differently to high temperatures suggests that different organisms have found different solutions to their specific metabolic and environmental challenges.

Pathogenicity is a complex phenotype combining cell-autonomous mechanisms with host-pathogen interactions. In addition to high-temperature growth, the ability to adhere to a substrate is a key mechanism of pathogenicity in *C. albicans* (71). Interestingly, *NCS2* and *NCS6* are upregulated during biofilm formation in *Candida* (72). Furthermore, genes required for wobble-uridine modifications have been identified in screens for modifiers of colony formation and have been shown to exhibit morphology phenotypes at the single cell level in baker's yeast (73,74). Consistent with this, we showed that a lack of U_34_ modification leads to morphology defects in *C. albicans* and a decreased ability to kill epithelial cells in culture (Figure 5C–E and Supplementary Figure S5C).

Wobble-uridine modifications tune codon-translation rates and are critical to ensure protein homeostasis (12,13,59). Consistent with this, we found that insoluble proteins were abundant in wild-type yeast grown at 37 °C while the levels of such protein aggregates were significantly lower in NCS2* yeast, consistent with findings in other tRNA modification mutants (13,59,74). The accumulation of protein aggregates reduces cellular fitness due to the loss of functional proteins, proteotoxic stress, the inability to clear misfolded proteins, and perturbations of amino acid homeostasis (75–77). This reduces the ability of cells to adapt to challenging environments. Upon infection, oxidative stress imposed by the host immune system and a high body temperature will perturb protein folding. High levels of tRNA modifications optimize translation and likely benefit the pathogen through more efficient translation. Protein homeostasis defects will not only affect the cytoplasmic proteome but also secreted cell-wall proteins, consistent with the observed sensitivity to CFW (Supplementary Figure S1B) as well as morphology phenotypes and a downregulation of cell-wall specific GO terms (Figures 5C, D, 6C, D, Supplementary Figure S5C and Supplementary Figure S6C, D).

Compared to baker's yeast, *C. albicans* appeared less affected by the absence of mcm^5^s^2^U. While the deletion of *NCS2* impaired high-temperature growth in *S. cerevisiae*, this was not the case for *C. albicans*. Interestingly, *C*. *albicans* has undergone a reassignment of the CTG codon during evolution leading to variable levels of amino acid misincorporation and a stochastical proteome (78,79). The cells are, therefore, more adapted to remedy protein-homeostasis defects also for the mistranslation of other codons (80). Nevertheless, we observed a perturbation of codon-specific translation, colony-morphology defects, and decreased virulence in hypomodified *C. albicans* and *S. cerevisiae*. Our work suggests that targeting tRNA modification enzymes to reduce modification levels is a promising strategy to treat pathogenic yeast infections. This approach is specific and acts orthogonal to current antifungal drugs (Supplementary Figure S5B). It is therefore an exciting prospect that drugging of tRNA modifications may give rise to novel antifungals.

## Supplementary Material

gkad564_Supplemental_File

## Data Availability

All oligonucleotides and plasmids used in this study are listed in Supplementary Table S3 and Supplementary table S4. The description of the algorithms applied are found in the methods section. Source codes for codon-occupancy calculations are available at (https://github.com/LeidelLab/Codon_occupancy_cal, permanent DOI: 10.5281/zenodo.8054266). The deep sequencing data from *C. albicans* ribosome profiling experiments are available in the Gene Expression Omnibus database under accession code GSE199422 except for wild-type *C. albicans* at 30 °C, which is available under accession code GSE136940.
