# Supplementary material for: Ncs2* mediates in vivo virulence of pathogenic yeast through sulphur modification of cytoplasmic transfer RNA
Source: Nucleic Acids Res. 2023 Jul 18;51(15):8133–49. doi: 10.1093/nar/gkad564 (PMC10450187; doi:10.1093/nar/gkad564)
Supplement: gkad564_Supplemental_File [file gkad564_supplemental_file.pdf]

Ncs2* mediates *in vivo* virulence of pathogenic yeast through sulphur modification of cytoplasmic transfer RNA

Fiona Alings[1], Karin Scharmann[1], Cristian Eggers[1,2,3], Bettina Böttcher[4,5], Mikołaj Sokołowski[6], Ekaterina Shvetsova[2,3], Puneet Sharma[1,2], Joël Roth[2], Leon Rashiti[2], Sebastian Glatt[6], Sascha Brunke[4], Sebastian A. Leidel[1,2,3,7*]

[1] Max Planck Research Group for RNA Biology, Max Planck Institute for Molecular Biomedicine, Muenster, Germany
[2] Department of Chemistry, Biochemistry and Pharmaceutical Sciences, University of Bern, Bern, Switzerland
[3] Graduate School for Cellular and Biomedical Sciences, University of Bern, Bern, Switzerland
[4] Department of Microbial Pathogenicity Mechanisms, Leibniz Institute for Natural Product Research and Infection Biology - Hans Knoell Institute, Jena, Germany
[5] Septomics Research Center, Friedrich Schiller University and Leibniz Institute for Natural Product Research and Infection Biology - Hans Knoell Institute, Jena, Germany
[6] Max Planck Research Group at the Malopolska Centre of Biotechnology, Jagiellonian University, Krakow, Poland
[7] Multidisciplinary Center for Infectious Diseases, University of Bern, Bern, Switzerland

* To whom correspondence should be addressed. Tel: +41 31 684 4296; Email: sebastian.leidel@unibe.ch

Supplementary Information

Supplementary Figure Legends

Supplementary Figure S1. *NCS2** affects yeast growth and tRNA 2-thiolation. (**A**) Schematic representation of the *NCS2* alleles used in this study. Upstream region (maroon), *NCS2* (blue), *HIS3*-selection cassette (orange/yellow), downstream region (grey). The red mark indicates the position of the A212T point mutation leading to H71L. (**B**) Spot-dilution assays using isogenic yeast strains on YPD (1 mg/ml paromomycin or 15 µg/ml calcofluor white (CFW)). (**C**, **D**) Northern-blot analysis of total RNA from wild type and *NCS2** yeast grown on YPD at 25 °C, 30 °C, and 37 °C. The probes are against $tRNA^{Gln}_{UUG}$ (**C**) and $tRNA^{Glu}_{UUC}$ (**D**). The upper gel contains ([*N*-acryloylamino]phenyl)mercuric chloride (APM). (**E**, **F**) Quantification of the fraction of s^2U-labeled tRNA of northern-blot replicates: (**E**) $tRNA^{Gln}_{UUG}$: *NCS2*: 58 % ± 1 % vs. *NCS2**: 58 % ± 4 % at 25 °C, P = 0.773; *NCS2*: 61 % ± 5 % vs. *NCS2**: 61 % ± 1 % at 30 °C, P = 0.934; *NCS2*: 32 % ± 5 % vs. *NCS2**: 56 % ± 1 at 37 °C, P = 0.001. (**F**) $tRNA^{Glu}_{UUC}$: *NCS2*: 67 % ± 7 % vs. *NCS2**: 63 % ± 5 % at 25 °C, P = 0.467; *NCS2*: 62 % ± 8 % vs. *NCS2**: 63 % ± 5 % at 30 °C, P = 0.719; *NCS2*: 40 % ± 7 % vs. *NCS2**: 60 % ± 5 % at 37 °C, P = 0.016. (n = 3). Data are represented as mean ± SEM. ** = P ≤ 0.01, * = P ≤ 0.05, ns = P > 0.05 (Two-sided student's t-test).

Supplementary Figure S2. *NCS2** modulates tRNA functionality. (**A-B**) Spot-dilution assay of *NCS2* and *NCS2** strains in combination with gene deletions that affect wobble uridine modifications grown on YPD. (**A**) *NCS2* and *NCS2** in combination with *elp4*Δ and *elp6*Δ (1 nM rapamycin or 1.4 mM diamide). (**B**) *NCS2ncs6*Δ and *NCS2*ncs6*Δ yeast (1 nM rapamycin).

Supplementary Figure S3. Molecular characterization of Ncs2. (**A**) Alignment of Ncs2 protein sequences from different species using T-coffee (1, 2) (for accession numbers see Methods). The colour of the amino acids indicates the likelihood that the residues are correctly aligned (blue: 0, dark red: 9). Values >5 (yellow, orange, and red) are likely correctly aligned. H71L is indicated in red. The conserved region bearing the mutation is highlighted by a black box. (**B**) Alphafold-based model of the Ncs2/6 heterodimer bound to tRNA in cartoon representation. Putative binding sites of ATP and the coordination of the iron/sulphur cluster have been indicated with ball-and-stick models. Zinc atoms (light brown) positioned in the N- and C-terminal domains of both Ncs2 and Ncs6 are enlarged for visibility (Ncs2: cyan, Ncs6: purple, tRNA: brown). (**C**) GST-pulldown assay for interaction of CtNcs2 and CtNcs6 (top: input, bottom: pulldown). (**D**) Thermal-shift assay for CtNcs2, CtNcs6 and CtNcs2/6 complex stability. Melting temperatures were calculated from the peaks of the first derivative. CtNcs2: 45.7 ± 0.1 °C; CtNcs6: 42.1 ± 0.3 °C; CtNcs2/6: 54.2 ± 0.0 °C. (**E-F**) Thermal-shift assay measuring the influence of nucleotides on CtNcs2 (**E**) and the CtNcs2/6 complex (**F**). (**E**) Melting temperatures for CtNcs2. No nucleotide: 44.5 ± 0.1 °C; + ATP: 53.6 ± 0.5 °C; + ADP: 53.7 ± 0.1 °C; + AMP-PNP: 51.1 ± 0.1 °C. (**F**) Melting temperatures for the CtNcs2/6 complex: no nucleotide: 54.6 ± 0.0 °C; + ATP: 57.3 ± 0.3 °C; + ADP: 56.3 ± 0.2 °C; + AMP-PNP: 57.7 ± 0.1 °C.

Supplementary Figure S4. *NCS2* modulates virulence in pathogenic baker's yeast. (**A**) Spot-dilution assays of wild type and *ncs2*Δ yeast derived from clinical isolates grown on YPD at different temperatures. (**B**) Northern-blot analysis of total RNA from YJM223-derived strains shown in Fig. 4B grown at different temperatures in YPD. The probe is against $tRNA^{Lys}_{UUU}$. The gel contains ([*N*-acryloylamino]phenyl)mercuric chloride (APM). The fraction of s^2U-labeled tRNA is indicated below. (**C**) Invasion assay of wild type and *ncs2*Δ yeast derived from clinical isolates YJM128 and YJM223. Strains were grown at 37 °C and spotted on YPD. After two days, the plates were washed, and strains allowed to grow for 24 h at 30 °C. Before wash (left), after re-growth (right).

Supplementary Figure S5. *NCS2* in *Candida albicans*. (**A**) Northern-blot analysis of tRNA from wild type *Candida albicans* grown at different temperatures in YPD. The probes are against $tRNA^{Glu}_{UUC}$ (left), $tRNA^{Gln}_{UUG}$ (middle), and $tRNA^{Lys}_{UUU}$ (right). The gels contain ([*N*-acryloylamino]phenyl)mercuric chloride (APM). The fraction of s^2U labelled tRNA is indicated below (n = 3). (**B**, **C**) Spot-dilution assays of *Candida albicans* strains grown on YPD. (**B**) Heterozygous and homozygous *ncs2*Δ mutants grown at 30 °C, 37 °C, and 41 °C (6 µM nystatin, 10 µg/ml fluconazole or 5 µM natamycin). (**C**) Different *NCS2* alleles grown on YPD at different temperatures.

Supplementary Figure S6. Translation defects in *Candida albicans*. (**A**-**D**) Gene ontology (GO) terms mis-regulated in *ncs2*Δ/Δ *C. albicans* relative to wild type grown in YPD at 30 °C and 41 °C. (**A**) and (**C**) Biological process. (**B**) and (**D**) Cellular component.

Supplementary Table S1. Summary of stress phenotypes of *ncs2*Δ, *NCS2*, and *NCS2** yeast in spotting assays at 37 °C. Red indicates sensitivity, yellow intermediate growth, and green resistance towards the tested stress. Light green indicates weak growth differences observed between *NCS2* and *NCS2**.

Supplementary Table S2. Yeast strains used in this study.

Supplementary Table S3. Northern probes and oligonucleotides used in this study.

Supplementary Table S4. Plasmids used in this study.

References:

1. Notredame,C., Higgins,D.G. and Heringa,J. (2000) T-coffee: a novel method for fast and accurate multiple sequence alignment. *Journal of Molecular Biology*, **302**, 205–217.

2. Di Tommaso,P., Moretti,S., Xenarios,I., Orobitg,M., Montanyola,A., Chang,J.-M., Taly,J.-F. and Notredame,C. (2011) T-Coffee: a web server for the multiple sequence alignment of protein and RNA sequences using structural information and homology extension. *Nucleic Acids Research*, **39**, W13–W17.

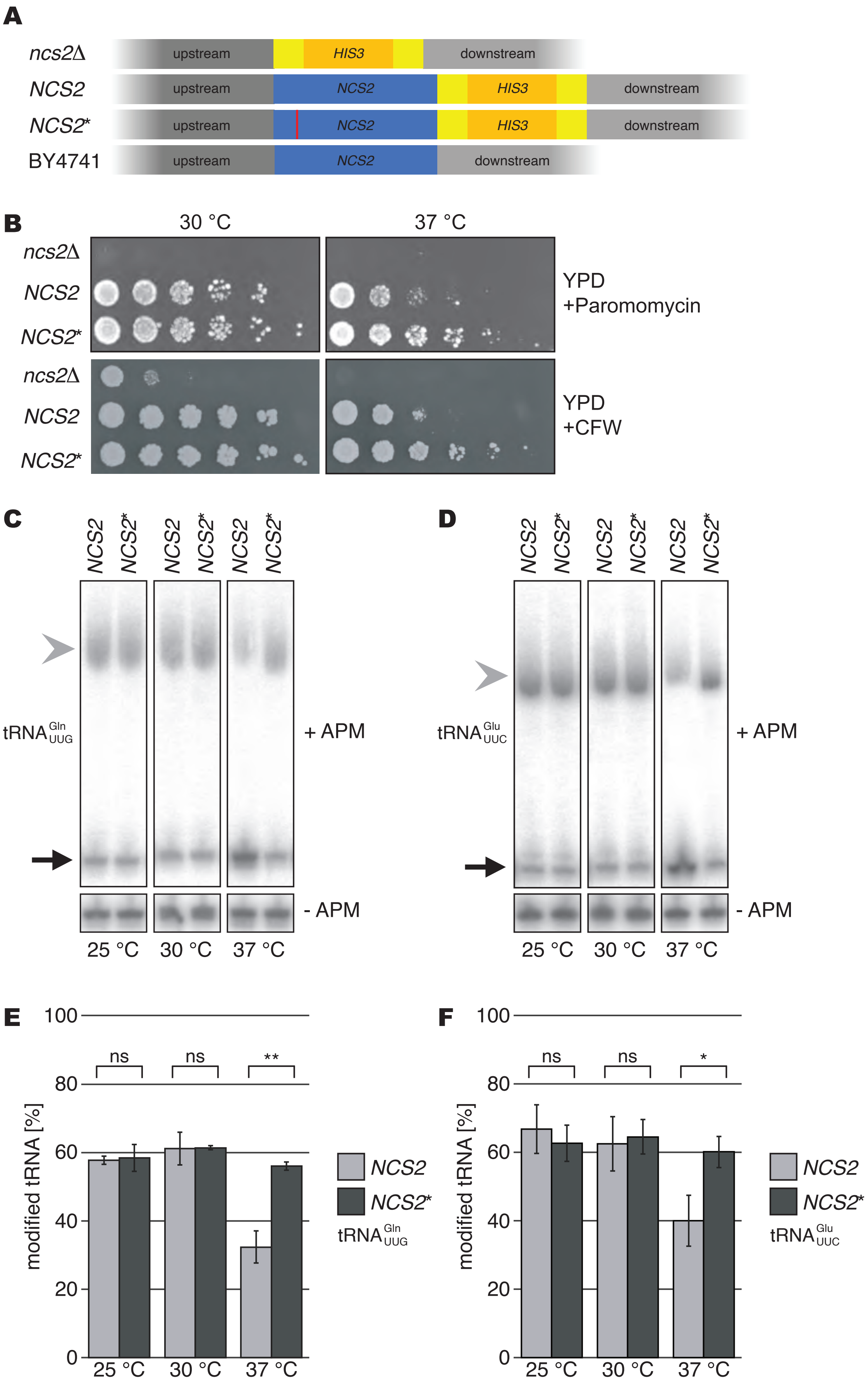

Supplementary Figure S1

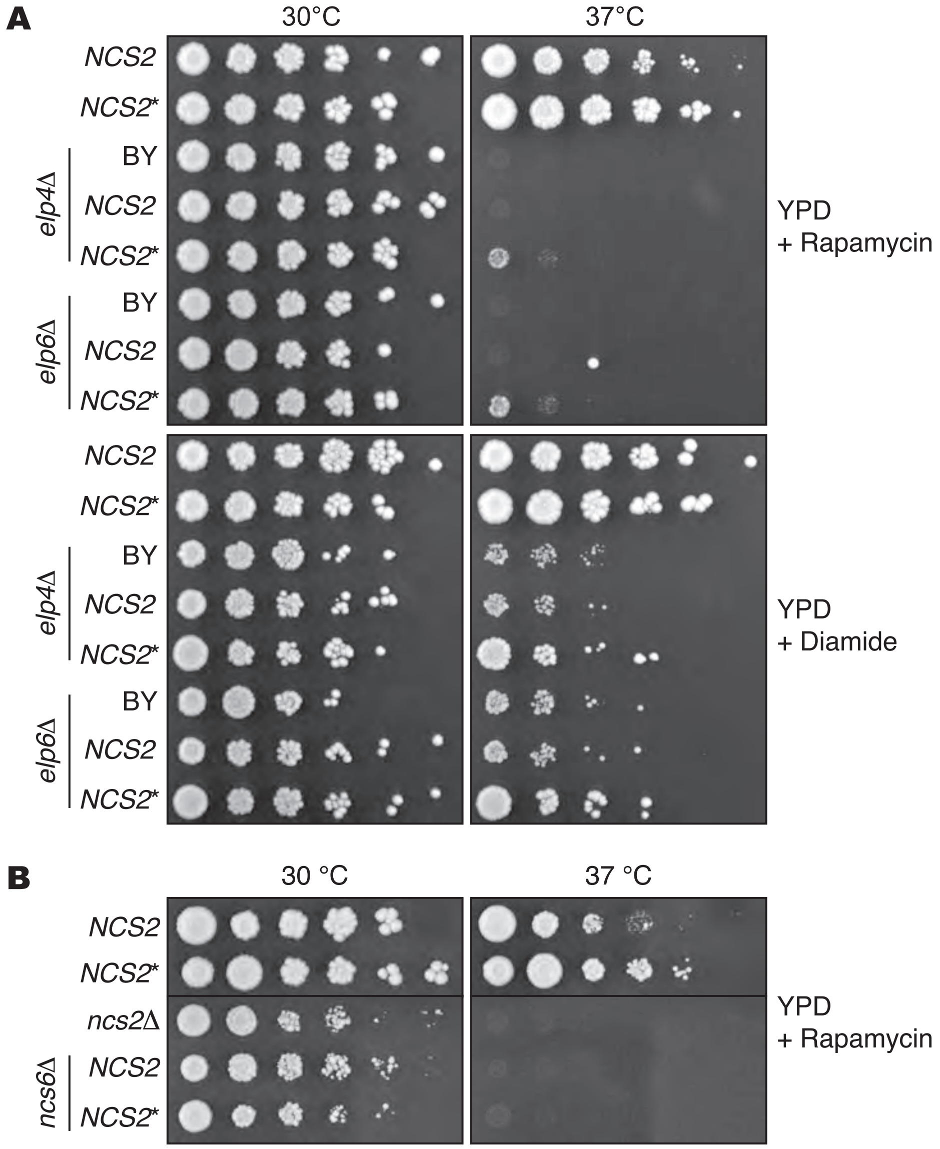

Supplementary Figure S2

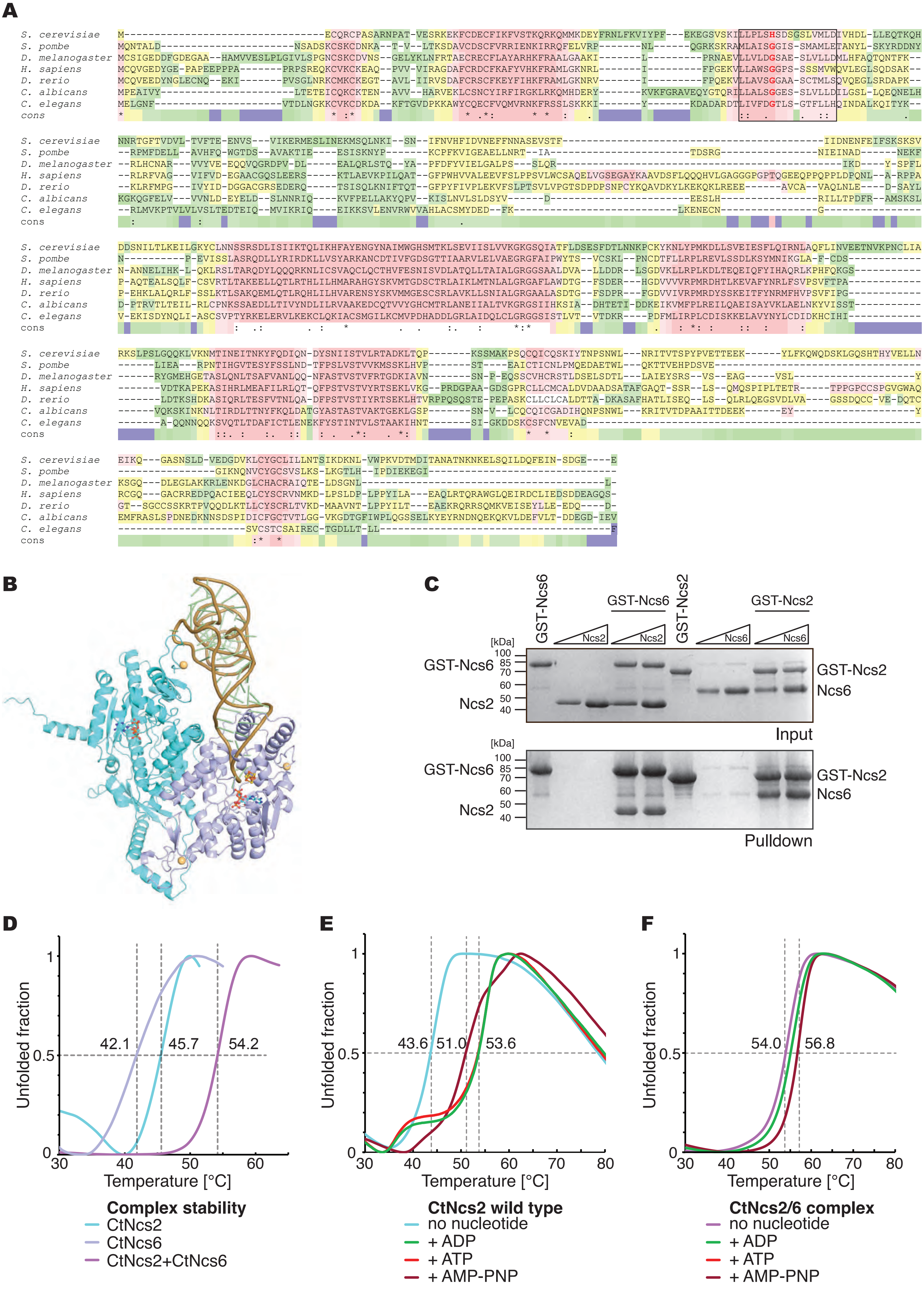

Supplementary Figure S3

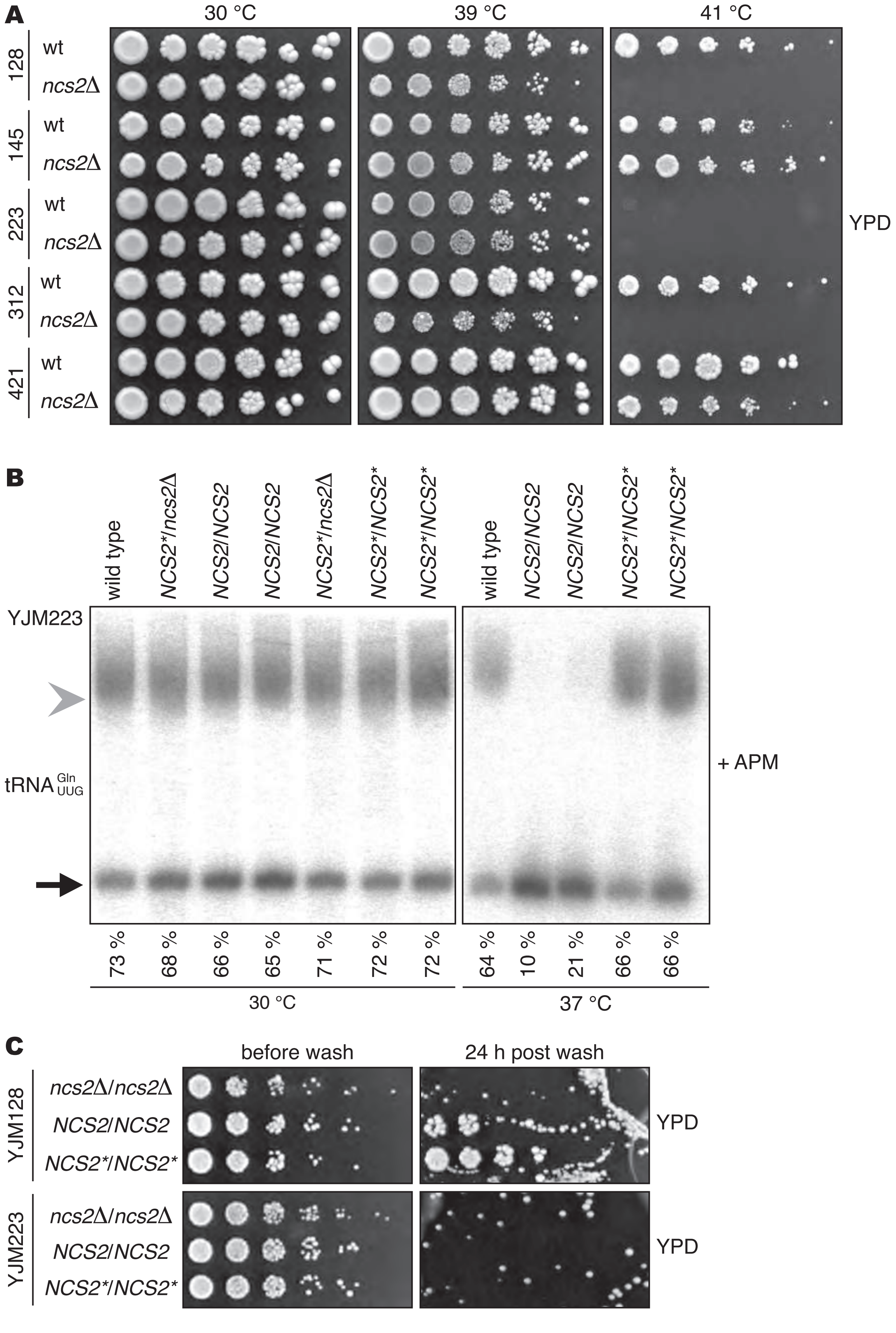

Supplementary Figure S4

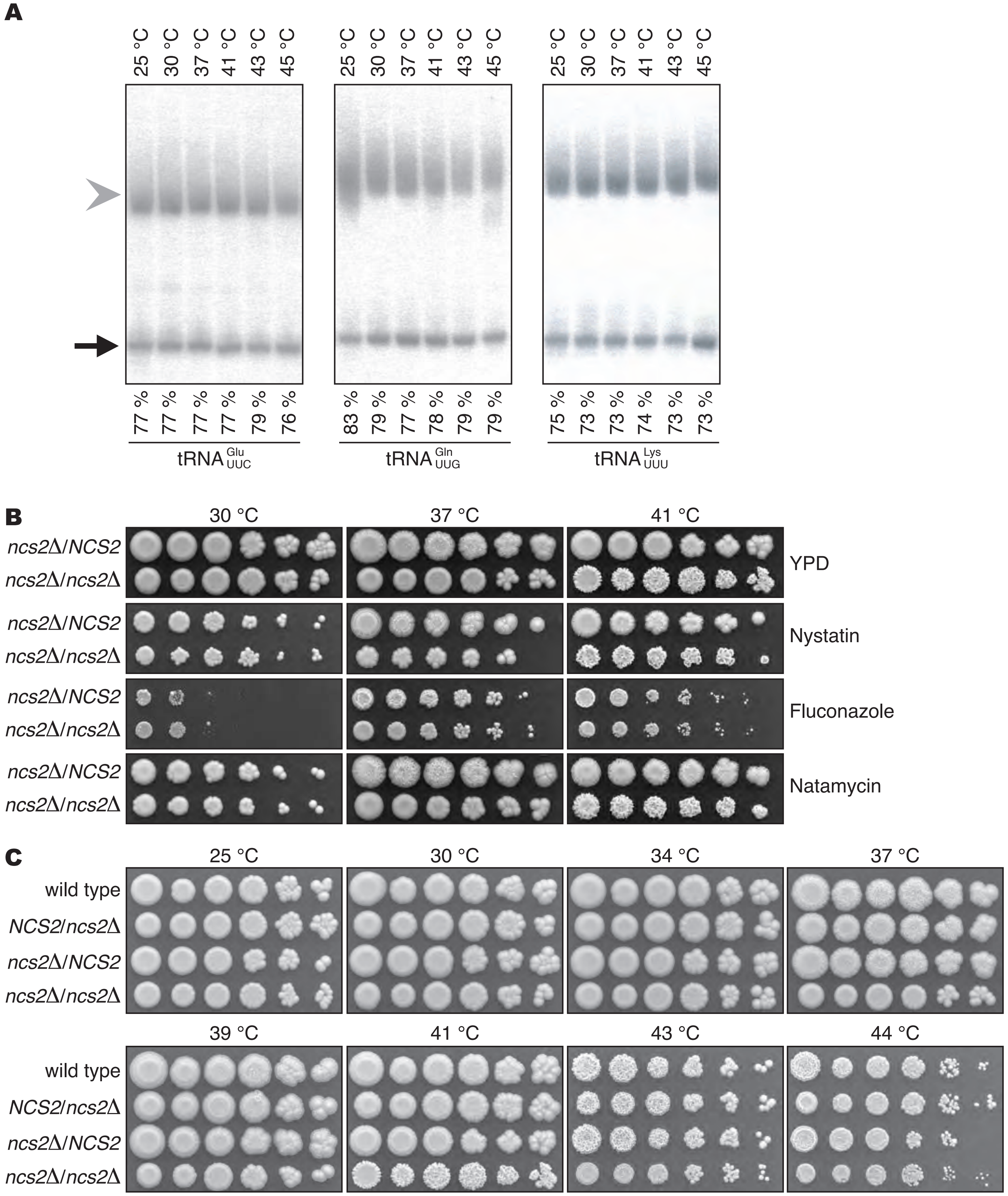

Supplementary Figure S5

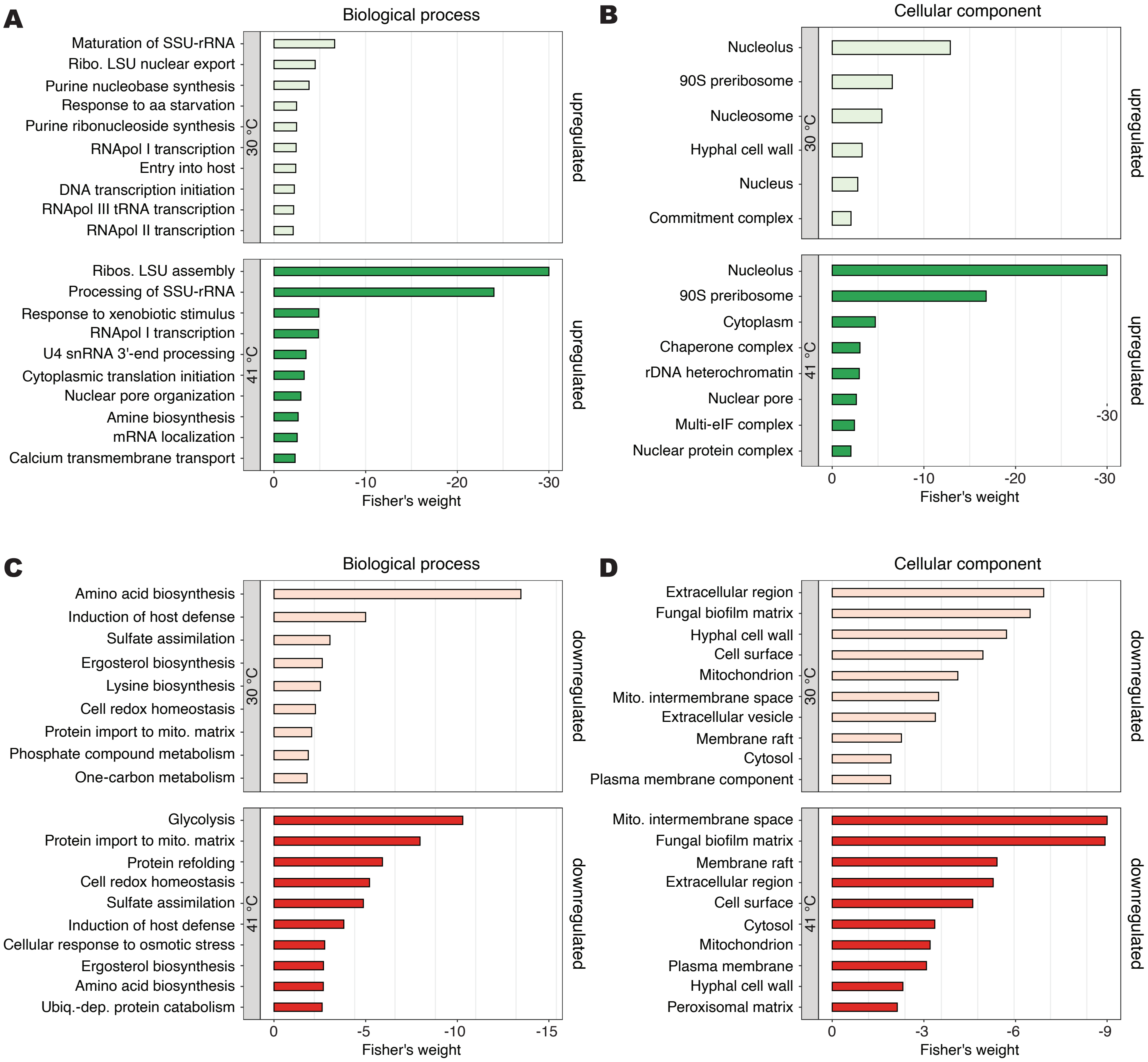

Supplementary Figure S6

**Drug**	**Range tested**	**Optimal concentration**	***ncs2D***	***NCS2***	***NCS2****
Rapamycin	2.5 - 3.0 nM	2.7 nM			
Caffeine	4 - 14 mM	6 mM			
Paromomycin	0.5 - 1.4 mg/ml	0.8 mg/ml			
Calcofluor white	2.5 - 50 µg/ml	15 µg/ml			
Methyl methanesulfonate	0.005 - 0.03 %	0.02 %			
Diamide	1.4 - 1.8 mM	1.6 mM			
Dimethyl sulfoxide	2 - 8 %	6 %			
Hydrogen peroxide	0.5 – 8 mM	6.5 mM			
Tert -butyl hydroperoxide	0.4 – 1.5 mM	1.1 mM			
Tithiothreitol	1.7 – 20 mM	20 mM			
b-mercaptoethanol	10 – 25 mM	22 mM			
Tunicamycin	0.25 – 1.4 µg/ml	1.2 µg/ml			
Sodium chloride	0.2 – 2 M	1.6 M			

Supplementary Table S1

Strain	Genotype	Background
wild type - BY4741	MAT a his3 Δ1 leu2 Δ0 met15 Δ0 ura3 Δ0	S288c
wild type - BY4742	MAT α his3 Δ1 leu2 Δ0 lys2 Δ0 ura3 Δ0	S288c
wild type - BY4743	MAT a/α his3 Δ1/his3 Δ1 leu2 Δ0/leu2 Δ0 LYS2/lys2 Δ0 met15 Δ0/MET15 ura3 Δ0/ura3 Δ0	S288c
wild type - Y1026	MAT a ura3 ::URA3 - LexAop - LacZ ura 3-1 leu2-3 ,-112 his3-11,-15 trp1-1 ade2-1 can1-100	W303
wild type - YJM128		clinical isolate (lung)
wild type - YJM145	Segregant of YJM128	clinical isolate
wild type - YJM223		clinical isolate (blood)
wild type - YJM312		clinical isolate (ascites fluid)
wild type - YJM421	Segregant of YJM419	clinical isolate
ncs2Δ	MAT a his3Δ1 leu2Δ0 met15Δ0 ura3Δ0 ncs2::kanMX	S288c
urm1Δ	MAT a his3Δ1 leu2Δ0 met15Δ0 ura3Δ0 urm1::kanMX	S288c
elp4Δ	MAT a his3Δ1 leu2Δ0 met15Δ0 ura3Δ0 elp4::kanMX	S288c
elp6Δ	MAT a his3Δ1 leu2Δ0 met15Δ0 ura3Δ0 elp6::kanMX	S288c
NCS2	MAT a his3Δ1 leu2Δ0 met15Δ0 ura3Δ0 NCS2::HIS3MX6	S288c
NCS2-H71L (NCS2*)	MAT a his3Δ1 leu2Δ0 met15Δ0 ura3Δ0 NCS2*::HIS3MX6	S288c
NCS2-TAP	MAT a his3Δ1 leu2Δ0 met15Δ0 ura3Δ0 NCS2-TAP::HIS3MX6	S288c
NCS2*-TAP	MAT a his3Δ1 leu2Δ0 met15Δ0 ura3Δ0 NCS2*-TAP::HIS3MX6	S288c
NCS2	MAT α his3Δ1 leu2Δ0 lys2Δ0 ura3Δ0 NCS2::HIS3MX6	S288c
NCS2*	MAT α his3Δ1 leu2Δ0 lys2Δ0 ura3Δ0 NCS2*::HIS3MX6	S288c
NCS2-TAP	MAT α his3Δ1 leu2Δ0 lys2Δ0 ura3Δ0 NCS2-TAP::HIS3MX6	S288c
NCS2*-TAP	MAT α his3Δ1 leu2Δ0 lys2Δ0 ura3Δ0 NCS2*-TAP::HIS3MX6	S288c
NCS2_H71A	MAT a his3Δ1 leu2Δ0 met15Δ0 ura3Δ0 NCS2_H71A::HIS3MX6	S288c
NCS2_H71I	MAT a his3Δ1 leu2Δ0 met15Δ0 ura3Δ0 NCS2_H71I::HIS3MX6	S288c
NCS2 ncs6Δ	MAT a his3Δ1 leu2Δ0 met15Δ0 ura3Δ0 NCS2::HIS3MX6 ncs6::kanMX	S288c
NCS2* ncs6Δ	MAT a his3Δ1 leu2Δ0 met15Δ0 ura3Δ0 NCS2*::HIS3MX6 ncs6::kanMX	S288c
NCS2 urm1Δ	MAT a his3Δ1 leu2Δ0 met15Δ0 ura3Δ0 NCS2::HIS3MX6 urm1::kanMX	S288c
NCS2* urm1Δ	MAT a his3Δ1 leu2Δ0 met15Δ0 ura3Δ0 NCS2*::HIS3MX6 urm1::kanMX	S288c
NCS2 elp4Δ	MAT a his3Δ1 leu2Δ0 met15Δ0 ura3Δ0 NCS2::HIS3MX6 elp4::kanMX	S288c
NCS2* elp4Δ	MAT a his3Δ1 leu2Δ0 met15Δ0 ura3Δ0 NCS2*::HIS3MX6 elp4::kanMX	S288c
NCS2 elp6Δ	MAT a his3Δ1 leu2Δ0 met15Δ0 ura3Δ0 NCS2::HIS3MX6 elp6::kanMX	S288c
NCS2* elp6Δ	MAT a his3Δ1 leu2Δ0 met15Δ0 ura3Δ0 NCS2*::HIS3MX6 elp6::kanMX	S288c
ncs2Δ /ncs2Δ	MAT a/a his3Δ1/his3Δ1 leu2Δ0/leu2Δ0 LYS2/lys2Δ0 met15Δ0/MET15 ura3Δ0/ura3Δ0 ncs2::kanMX/ncs2::NatMX	S288c
ncs2Δ /NCS2	MAT a/a his3Δ1/his3Δ1 leu2Δ0/leu2Δ0 LYS2/lys2Δ0 met15Δ0/MET15 ura3Δ0/ura3Δ0 ncs2::kanMX/ncs2::NatMX	S288c
ncs2Δ /NCS2*	MAT a/a his3Δ1/his3Δ1 leu2Δ0/leu2Δ0 LYS2/lys2Δ0 met15Δ0/MET15 ura3Δ0/ura3Δ0 ncs2::kanMX/ncs2::NatMX	S288c
NCS2 /NCS2	MAT a/a his3Δ1/his3Δ1 leu2Δ0/leu2Δ0 LYS2/lys2Δ0 met15Δ0/MET15 ura3Δ0/ura3Δ0 NCS2::kanMX/NCS2::NatMX	S288c
NCS2 /NCS2*	MAT a/a his3Δ1/his3Δ1 leu2Δ0/leu2Δ0 LYS2/lys2Δ0 met15Δ0/MET15 ura3Δ0/ura3Δ0 NCS2::kanMX/NCS2*::NatMX	S288c
NCS2* /NCS2*	MAT a/a his3Δ1/his3Δ1 leu2Δ0/leu2Δ0 LYS2/lys2Δ0 met15Δ0/MET15 ura3Δ0/ura3Δ0 NCS2*::kanMX/NCS2*::NatMX	S288c
ncs2Δ /ncs2Δ	MAT a/a his3Δ1/his3Δ1 leu2Δ0/leu2Δ0 LYS2/lys2Δ0 met15Δ0/MET15 ura3Δ0/ura3Δ0 ncs2::kanMX/ncs2::NatMX	YJM128
NCS2/ncs2Δ	MAT a/a his3Δ1/his3Δ1 leu2Δ0/leu2Δ0 LYS2/lys2Δ0 met15Δ0/MET15 ura3Δ0/ura3Δ0 NCS2::hphMX4/ncs2::NatMX	YJM128
NCS2*/ncs2Δ	MAT a/a his3Δ1/his3Δ1 leu2Δ0/leu2Δ0 LYS2/lys2Δ0 met15Δ0/MET15 ura3Δ0/ura3Δ0 NCS2*::hphMX4/ncs2::NatMX	YJM128
NCS2 /NCS2	MAT a/a his3Δ1/his3Δ1 leu2Δ0/leu2Δ0 LYS2/lys2Δ0 met15Δ0/MET15 ura3Δ0/ura3Δ0 NCS2::kanMX/NCS2::hphMX4	YJM128
NCS2* /NCS2*	MAT a/a his3Δ1/his3Δ1 leu2Δ0/leu2Δ0 LYS2/lys2Δ0 met15Δ0/MET15 ura3Δ0/ura3Δ0 NCS2*::kanMX/NCS2*::hphMX4	YJM128
ncs2Δ /ncs2Δ	MAT a/a his3Δ1/his3Δ1 leu2Δ0/leu2Δ0 LYS2/lys2Δ0 met15Δ0/MET15 ura3Δ0/ura3Δ0 ncs2::kanMX/ncs2::NatMX	YJM145
NCS2/ncs2Δ	MAT a/a his3Δ1/his3Δ1 leu2Δ0/leu2Δ0 LYS2/lys2Δ0 met15Δ0/MET15 ura3Δ0/ura3Δ0 NCS2::hphMX4/ncs2::NatMX	YJM145
NCS2*/ncs2Δ	MAT a/a his3Δ1/his3Δ1 leu2Δ0/leu2Δ0 LYS2/lys2Δ0 met15Δ0/MET15 ura3Δ0/ura3Δ0 NCS2*::hphMX4/ncs2::NatMX	YJM145
ncs2Δ /ncs2Δ	MAT a/a his3Δ1/his3Δ1 leu2Δ0/leu2Δ0 LYS2/lys2Δ0 met15Δ0/MET15 ura3Δ0/ura3Δ0 ncs2::kanMX/ncs2::NatMX	YJM223
NCS2/ncs2Δ	MAT a/a his3Δ1/his3Δ1 leu2Δ0/leu2Δ0 LYS2/lys2Δ0 met15Δ0/MET15 ura3Δ0/ura3Δ0 NCS2::hphMX4/ncs2::NatMX	YJM223
NCS2*/ncs2Δ	MAT a/a his3Δ1/his3Δ1 leu2Δ0/leu2Δ0 LYS2/lys2Δ0 met15Δ0/MET15 ura3Δ0/ura3Δ0 NCS2*::hphMX4/ncs2::NatMX	YJM223
NCS2 /NCS2	MAT a/a his3Δ1/his3Δ1 leu2Δ0/leu2Δ0 LYS2/lys2Δ0 met15Δ0/MET15 ura3Δ0/ura3Δ0 NCS2::kanMX/NCS2::hphMX4	YJM223
NCS2* /NCS2*	MAT a/a his3Δ1/his3Δ1 leu2Δ0/leu2Δ0 LYS2/lys2Δ0 met15Δ0/MET15 ura3Δ0/ura3Δ0 NCS2*::kanMX/NCS2*::hphMX4	YJM223
ncs2Δ /ncs2Δ	MAT a/a his3Δ1/his3Δ1 leu2Δ0/leu2Δ0 LYS2/lys2Δ0 met15Δ0/MET15 ura3Δ0/ura3Δ0 ncs2::kanMX/ncs2::NatMX	YJM312
NCS2/ncs2Δ	MAT a/a his3Δ1/his3Δ1 leu2Δ0/leu2Δ0 LYS2/lys2Δ0 met15Δ0/MET15 ura3Δ0/ura3Δ0 NCS2::hphMX4/ncs2::NatMX	YJM312
NCS2*/ncs2Δ	MAT a/a his3Δ1/his3Δ1 leu2Δ0/leu2Δ0 LYS2/lys2Δ0 met15Δ0/MET15 ura3Δ0/ura3Δ0 NCS2*::hphMX4/ncs2::NatMX	YJM312
ncs2Δ /ncs2Δ	MAT a/a his3Δ1/his3Δ1 leu2Δ0/leu2Δ0 LYS2/lys2Δ0 met15Δ0/MET15 ura3Δ0/ura3Δ0 ncs2::kanMX/ncs2::NatMX	YJM421
NCS2/ncs2Δ	MAT a/a his3Δ1/his3Δ1 leu2Δ0/leu2Δ0 LYS2/lys2Δ0 met15Δ0/MET15 ura3Δ0/ura3Δ0 NCS2::hphMX4/ncs2::NatMX	YJM421
NCS2*/ncs2Δ	MAT a/a his3Δ1/his3Δ1 leu2Δ0/leu2Δ0 LYS2/lys2Δ0 met15Δ0/MET15 ura3Δ0/ura3Δ0 NCS2*::hphMX4/ncs2::NatMX	YJM421
3HA-NCS6 NCS2-TAP	MAT α his3Δ1 leu2Δ0 lys2Δ0 ura3Δ0 NCS2-TAP::HIS3MX6 3HA-NCS6::URA	S288c
3HA-NCS6 NCS2*-TAP	MAT α his3Δ1 leu2Δ0 lys2Δ0 ura3Δ0 NCS2*-TAP::HIS3MX6 HA-NCS6::URA	S288c
FLAG-NCS6 NCS2-TAP	MAT α his3Δ1 leu2Δ0 lys2Δ0 ura3Δ0 NCS2-TAP::HIS3MX6 FLAG-NCS6::URA	S288c
FLAG-NCS6 NCS2*-TAP	MAT α his3Δ1 leu2Δ0 lys2Δ0 ura3Δ0 NCS2*-TAP::HIS3MX6 FLAG-NCS6::URA	S288c
wild type - SN87	leu2Δ/leu2Δ his1Δ/his1Δ URA3/ura3Δ::imm434 IRO1/iro1Δ::imm 434	SC5314
NCS2/ncs2Δ	leu2Δ/leu2Δ his1Δ/his1Δ URA3/ura3Δ::imm434 IRO1/iro1Δ::imm 434 ncs2::HIS1/NCS	SN87
NCS2/ncs2Δ	leu2Δ/leu2Δ his1Δ/his1Δ URA3/ura3Δ::imm434 IRO1/iro1Δ::imm 434 ncs2::LEU2/NCS	SN87
ncs2Δ /ncs2Δ	leu2Δ/leu2Δ his1Δ/his1Δ URA3/ura3Δ::imm434 IRO1/iro1Δ::imm 434 ncs2::LEU2/ncs2::HIS1	SN87

Supplementary Table S2

Designation	Parental vector	Gene of interest	Yeast marker	Bacterial marker	Comment	Source reference
pSZ229	pGR	*NCS2*	His	amp	For yeast transformation	This study
pSZ230	pGR	*NCS2**	His	amp	For yeast transformation	This study
pSZ204	pGR	*NCS2* -TAP	His	amp	For yeast transformation	This study
pSZ205	pGR	*NCS2** -TAP	His	amp	For yeast transformation	This study
pSZ229_H71A	pGR	*ncs2* _H71A	His	amp	For yeast transformation	This study
pSZ229_H71I	pGR	*ncs2* _H71I	His	amp	For yeast transformation	This study
pSZ229_hph	pGR	*NCS2*	hph	amp	For yeast transformation	This study
pSZ230_hph	pGR	*NCS2**	hph	amp	For yeast transformation	This study
pAG32		hph	hph	amp	Template to amplify hphMX4 cassette	Goldstein & McCusker, 1999
pFA6a-natNT2		nat	nat	amp	Template to amplify Nat-ADH cassette	Janke et al., 2004
pSZ10	pJG4-6	empty control	Trp	amp	Yeast-two hybrid vector	Leidel et al., 2009
pSZ09	pEG203	empty control	His	amp	Yeast-two hybrid vector	Leidel et al., 2009
pSZ94	pJG4-6	*NCS2*	Trp	amp	Yeast-two hybrid vector	This study
pSZ95	pEG203	*NCS2*	His	amp	Yeast-two hybrid vector	This study
pSZ96	pJG4-6	*NCS6*	Trp	amp	Yeast-two hybrid vector	This study
pSZ97	pEG203	*NCS6*	His	amp	Yeast-two hybrid vector	This study
pSZ207	pJG4-6	*NCS2**	Trp	amp	Yeast-two hybrid vector	This study
pSZ208	pEG203	*NCS2**	His	amp	Yeast-two hybrid vector	This study
pSZ94_H71A	pJG4-6	*ncs2* _H71A	Trp	amp	Yeast-two hybrid vector	This study
pSZ95_H71A	pEG203	*ncs2* _H71A	His	amp	Yeast-two hybrid vector	This study
pSZ94_H71I	pJG4-6	*ncs2* _H71I	Trp	amp	Yeast-two hybrid vector	This study
pSZ95_H71I	pEG203	*ncs2* _H71I	His	amp	Yeast-two hybrid vector	This study
CtNcs2-pETM30	pETM30	CtNcs2	---	kan	Protein expression	H. Besir, G. Stein
CtNcs6-pETM30	pETM30	CtNcs6	---	kan	Protein expression	H. Besir, G. Stein
CIp10HL	pETM30	*HIS1 LEU2*	---	kan	For yeast transformation	

Supplementary Table S3

Designation	Name	Sequence	Comment	Species	Source/Reference
MS0394	ncs2_H71A_L	CTGCTCCCCTTATCAGCTTCTGATTCAGGATCTCTGG	Site directed mutatgenesis	*Saccharomcyces cerevisiae*	This study
MS0395	ncs2_H71A_R	CCAGAGATCCTGAATCAGAAGCTGATAAGGGGAGCAG	Site directed mutatgenesis	*Saccharomcyces cerevisiae*	This study
MS0693	ncs2_His71Ile_L	CTGCTCCCCTTATCAATTTCTGATTCAGGATCTCTGG	Site directed mutatgenesis	*Saccharomcyces cerevisiae*	This study
MS0694	ncs2_His71Ile_R	CCAGAGATCCTGAATCAGAAATTGATAAGGGGAGCAG	Site directed mutatgenesis	*Saccharomcyces cerevisiae*	This study
MS0015	ncs2det_L	TGGTGAGTGGTGGAGTTCCT	Verification of gene deletion	*Saccharomcyces cerevisiae*	This study
MS0016	ncs2det_R	CGCAACGACGCTACAAATAA	Verification of gene deletion	*Saccharomcyces cerevisiae*	This study
MS0039	ncs2det_R2	GGTGGACCGTCAAGTGAAAT	Verification of gene deletion	*Saccharomcyces cerevisiae*	This study
MS0050	Urm1D_L	CCATCAAATGCCATCAGTTCT	Amplification for gene deletion	*Saccharomcyces cerevisiae*	This study
MS0051	Urm1D_R	CACTTCCCGTCCTTCTCTTG	Amplification for gene deletion	*Saccharomcyces cerevisiae*	This study
MS0066	TefTerm_L	TGGTCGCTATACTGCTGCTG	Verification of gene deletion	*Saccharomcyces cerevisiae*	This study
MS0070	urm1_farout_L	CCACATGAGCGGTATACGAA	Verification of gene deletion	*Saccharomcyces cerevisiae*	This study
MS0074	Kan_R	GATGGTCGGAAGAGGCATAA	Verification of gene deletion	*Saccharomcyces cerevisiae*	This study
MS0081	Ncs6_Duet2L	cgcgGgatccaTCGTTTACTGCTCCTTCAGATCC	Verification of gene deletion	*Saccharomcyces cerevisiae*	This study
MS0135	ncs2_R (XhoI)	gcgctcgagTTATTCCTCCCCATCGGAAT	Verification of gene deletion	*Saccharomcyces cerevisiae*	This study
MS0136	ncs2_int_R	CAGAATGTGATAAGGGGAGCA	Verification of gene deletion	*Saccharomcyces cerevisiae*	This study
MS0171	ncs2_L(EcoRI)	cgcgaattcGAGTGCCAGAGGTGCCC	Verification of gene deletion	*Saccharomcyces cerevisiae*	This study
MS0201	Ncs2-detect2-L	GGATTTGATGGGGCTGTAGA	Verification of gene deletion	*Saccharomcyces cerevisiae*	This study
MS0220	Nat_R2	AGCCGTGTCGTCAAGAGTG	Verification of gene deletion	*Saccharomcyces cerevisiae*	This study
MS0227	Tef PromR	CTGCAGCGAGGAGCCGTAAT	Verification of gene deletion	*Saccharomcyces cerevisiae*	This study
MS0228	Ncs2_int_L	CGATGTTGAAGACGGTGATG	Verification of gene deletion	*Saccharomcyces cerevisiae*	This study
MS0229	His3+_L	TGTATCGGTGACCTGCACAT	Verification of gene deletion	*Saccharomcyces cerevisiae*	This study
MS0230	His3+_R	TGTGCAGGTCACCGATACAT	Verification of gene deletion	*Saccharomcyces cerevisiae*	This study
MS0403	Ncs6_det_R	CAGTTCGGTAAGCCAGTGGT	Verification of gene deletion	*Saccharomcyces cerevisiae*	This study
MS0406	Nat_cass_L	GCTTCGTGGTCGTCTCGTA	Verification of gene deletion	*Saccharomcyces cerevisiae*	This study
MS0407	Nat_cass_R	CAGGGCATGCTCATGTAGAG	Verification of gene deletion	*Saccharomcyces cerevisiae*	This study
MS0408	G418_det_L	CCTCTTCCGACCATCAAGC	Verification of gene deletion	*Saccharomcyces cerevisiae*	This study
MS0409	G418_det_R	GGCAGTTCCATAGGATGGC	Verification of gene deletion	*Saccharomcyces cerevisiae*	This study
MS0446	TefProm_L	AGGATTTGCCACTGAGGTTC	Verification of gene deletion	*Saccharomcyces cerevisiae*	This study
MS0447	TefTerm_R	CGCACTTAACTTCGCATCTG	Verification of gene deletion	*Saccharomcyces cerevisiae*	This study
MS0561	ncs2delta_L	GGTCGTCCAAACACAAAAGG	Amplification for gene deletion	*Saccharomcyces cerevisiae*	This study
MS0562	ncs2delta_R	ACAGCAGCGCCTTCAATAAT	Amplification for gene deletion	*Saccharomcyces cerevisiae*	This study
MS0959	ncs6_delta_L	CGGAAGTTCAAGGAAGAACG	Amplification for gene deletion	*Saccharomcyces cerevisiae*	This study
MS0960	ncs2_delta_R	AGACGTGATGGCCCTGTAAC	Amplification for gene deletion	*Saccharomcyces cerevisiae*	This study
MS1096	Urm1_faroutR	TAGTACCCGGTAGCCCTTCA	Verification of gene deletion	*Saccharomcyces cerevisiae*	This study
MS1097	urm1genomic-L	GCTCAAAAATTCCCAAAGCA	Verification of gene deletion	*Saccharomcyces cerevisiae*	This study
MS1098	ncs6_det_L	CGCTCTATTGAGGTCCTTGC	Verification of gene deletion	*Saccharomcyces cerevisiae*	This study
MS1162	Nat CassL2	CTCTACATGAGCATGCCCTG	Verification of gene deletion	*Saccharomcyces cerevisiae*	This study
MS1173	NatL2	GAGGTCACCAACGTCAACG	Verification of gene deletion	*Saccharomcyces cerevisiae*	This study
MS1174	Nat2R	GGTAAGCCGTGTCGTCAAGA	Verification of gene deletion	*Saccharomcyces cerevisiae*	This study
MS1337	hph-check_R	AAACTTCTCGACAGACGTCG	Verification of gene deletion	*Saccharomcyces cerevisiae*	This study
MS1338	hph-check_L	GACGGCAATTTCGATGATGC	Verification of gene deletion	*Saccharomcyces cerevisiae*	This study
SZ4631	Urm1-Ggdelta(Xho)-R	cgctcgagttaATGTAATGTTGAAGTAAAAGAGATGAT	Verification of gene deletion	*Saccharomcyces cerevisiae*	This study
SZ4630	ncs6_R(XhoI)	gcgctcgagTCAAAAGCTGAGTTTCTCAAGAG	Verification of gene deletion	*Saccharomcyces cerevisiae*	This study
MS1183	primer1_ncs2_ko_C.a.	AATAGTGACAATACTCTCTG	Amplification for gene deletion	*Candida albicans*	This study
MS1184	primer3_ncs2_ko_C.a.	cacggcgcgcctagcagcggGATGAGTAGATGATGTAGATG	Amplification for gene deletion	*Candida albicans*	This study
MS1185	primer4_ncs2_ko_C.a.	gtcagcggccgcatccctgcGGAAATTATATAGTCAGACGAA	Amplification for gene deletion	*Candida albicans*	This study
MS1186	primer6_ncs2_ko_C.a.	GTTTGAGGAGTACATTTGTTA	Amplification for gene deletion	*Candida albicans*	This study
MS1187	univ. Primer 2_ko_C.a.	ccgctgctaggcgcgccgtgACCAGTGTGATGGATATCTGC	Amplification for gene deletion	*Candida albicans*	This study
MS1188	univ. Primer 5_ko_C.a.	gcagggatgcggccgctgacAGCTCGGATCCACTAGTAACG	Amplification for gene deletion	*Candida albicans*	This study
MS1189	ncs2 upstream check	GTTATTTTTTGCTCGAAACG	Verification of gene deletion	*Candida albicans*	This study
MS1190	ncs2 downstream check	GGTGAACAAGAACACATAAA	Verification of gene deletion	*Candida albicans*	This study
MS1191	HIS1 Left	ATTAGATACGTTGGTGGTTC	Verification of gene deletion	*Candida albicans*	This study
MS1992	HIS1 Right	AACACAACTGCACAATCTGG	Verification of gene deletion	*Candida albicans*	This study
MS1993	LEU2 Left	AGAATTCCCAACTTTGTCTG	Verification of gene deletion	*Candida albicans*	This study
MS1994	LEU2 Right	AAACTTTGAACCCGGCTGCG	Verification of gene deletion	*Candida albicans*	This study
MS1313	ncs2 downstream check	TTTATGTGTTCTTGTTCACC	Verification of gene deletion	*Candida albicans*	This study
MS1314	N2 upstr. Check p2	AAGACGACATTAACGACTGG	Verification of gene deletion	*Candida albicans*	This study
MS1315	N2 downst. Check p2	GAATATGCCGATAGTGGAGG	Verification of gene deletion	*Candida albicans*	This study
MP4910	tEUUC	TGGCTCCGATACGGGGAGTCGAAC	Northern probe for *S. cerevisiae*	*Saccharomcyces cerevisiae*	Leidel et al., 2009
MP4917	tKUUU	CTCCTCATAGGGGGCTCGAACCC	Northern probe for *S. cerevisiae*	*Saccharomcyces cerevisiae*	Leidel et al., 2009
MP4918	tQUUG	AGGTCCTACCCGGATTCGAACCGG	Northern probe for *S. cerevisiae*	*Saccharomcyces cerevisiae*	Leidel et al., 2009
MS1240	tEUUC	CTCCAATGCGGGGAATCGAACCC	Northern probe for *C. albicans*	*Candida albicans*	This study
MS1241	tKUUU	CGCTCTAAGCCGATTGAGCTAGCT	Northern probe for *C. albicans*	*Candida albicans*	This study
MS1242	tQUUG	AGGTCCTATCCGGATTCGAACCG	Northern probe for *C. albicans*	*Candida albicans*	This study

Supplementary Table S4
